# Neutrophil-derived Oxidants and Proteinases as Immunomodulatory Mediators in Inflammation

**DOI:** 10.1155/S0962935194000360

**Published:** 1994

**Authors:** V. Witko-Sarsat, B. Descamps-Latscha

**Affiliations:** INSERM U25 Hôpital Necker 161 rue de Sèvres Paris 75743 France

## Abstract

Neutrophils generate potent microbicidal molecules via the oxygen-dependent pathway, leading to the generation of reactive oxygen intermediates (ROI), and via the non-oxygen dependent pathway, consisting in the release of serine proteinases and metalloproteinases stored in granules. Over the past years, the concept has emerged that both ROI and proteinases can be viewed as mediators able to modulate neutrophil responses as well as the whole inflammatory process. This is well illustrated by the oxidative regulation of proteinase activity showing that oxidants and proteinases acts is concert to optimize the microbicidal activity and to damage host tissues. ROI and proteinases can modify the activity of several proteins involved in the control of inflammatory process. Among them, tumour necrosis factor-α and interleukin-8, are elective targets for such a modulation. Moreover, ROI and proteinases are also able to modulate the adhesion process of neutrophils to endothelial cells, which is a critical step in the inflammatory process.


Mediators of Inflammation 3, 257–273 (1994)

### Neutrophil-derived oxidants and proteinases as immunomodulatory mediators in inflammation

V. Witko-Sarsat
and B. Deşcamps-Latscha[CA]

INSERM U25, Hôpital Necker, 161 rue de Sèvres, 75743, Paris, France

[CA]Corresponding Author

**Neutrophils generate potent microbicidal molecules via the oxygen-dependent pathway, leading to the generation of reactive oxygen intermediates (ROI), and via the non-oxygen dependent pathway, consisting in the release of serine proteinases and metalloproteinases stored in granules. Over the past years, the concept has emerged that both ROI and proteinases can be viewed as mediators able to modulate neutrophil responses as well as the whole inflammatory process. This is well illustrated by the oxidative regulation of proteinase activity showing that oxidants and proteinases acts is concert to optimize the microbicidal activity and to damage host tissues. ROI and proteinases can modify the activity of several proteins involved in the control of inflammatory process. Among them, tumour necrosis factor-α and interleukin-8, are elective targets for such a modulation. Moreover, ROI and proteinases are also able to modulate the adhesion process of neutrophils to endothelial cells, which is a critical step in the inflammatory process.**

**Key words:** Adhesion molecules, IL-8, Myeloperoxidase, Neutrophil, Oxidants, Oxidative burst, Phagocyte, Proteinases, TNFα.

### Introduction

The aim of this report is to review our current understanding of radical oxygen intermediates (ROI) and proteinases in the context of a modulatory role of neutrophils in the inflammatory process. We will also review studies showing that besides their usual microbicidal role these molecules have important regulatory functions. The notion that inflammation is the net result of pro- and contra-inflammatory pathways[1] is well illustrated by ROI and proteinases which either alone, or in concert, may interact in up or down regulating the major inflammatory process. Added to the intrinsic complexity of the subject is the diversity of effects that can be mediated by ROI and proteinases, either on the phagocyte itself or on the target cells.

### Neutrophil-derived oxidants and proteinases

Neutrophils react to invading microorganisms and inflammatory mediators with a variety of coordinated responses, such as motility in response to chemotactic agents, cytoskeletal rearrangement, phagocytosis, and production of toxic mediators to allow the neutrophil to destroy pathogens. Although critical to host defence, neutrophils can damage normal cells and dissolve connective tissues by the release of a complex assortment of deleterious agents, including ROI and proteinases, leading to inflammatory disorders.[2,3]

Conventionally, two microbicidal pathways are defined, depending on whether or not they require oxygen. The oxygen dependent pathway depends on ROI whose production follows the activation of NADPH oxidase; the non-oxygen-dependent pathway reflects the actions of preformed enzymes and antimicrobial proteins stored in the neutrophil cytoplasmic granules and released upon activation as illustrated in Fig. 1.

Oxidative metabolism activation, known as the respiratory burst, first involves NADPH oxidase, which is an enzymatic complex composed of cytosolic and membrane proteins which ultimately translocate to the plasma membrane, leading to the generation of superoxide anion ($O_2^-$). The dismutation of $O_2^-$ can generate hydrogen peroxide ($H_2O_2$). Myeloperoxidase (MPO), an enzyme contained in azurophilic granules, in the presence of chloride and $H_2O_2$, catalyses the formation of potent chlorinated oxidants such as hypochlorous acid (HOCl) and chloramines, the so-called long-lived oxidants.[4–8] Much of what is known about the NADPH oxidase has come from studies of patients deficient in the system, who have chronic granulomatous disease (CGD). Owing to a genetic defect in the major component of the oxidase,

© 1994 Rapid Communications of Oxford Ltd

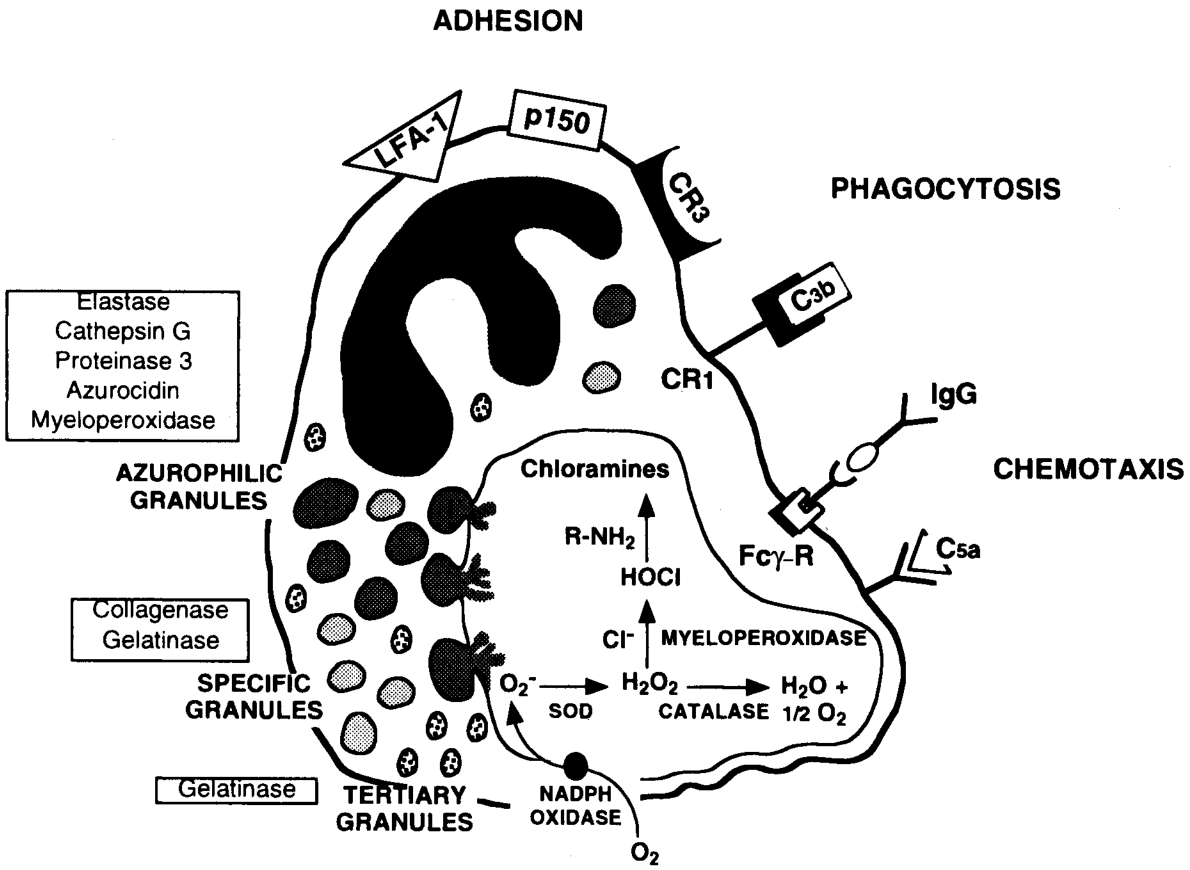

FIG 1. Neutrophil effector mechanisms involved in the defence against pathogens and in the modulation of inflammatory process. Neutrophils respond to inflammatory agents by expressing membrane receptors appropriate to the stimulation and by developing coordinated responses such as spreading, diapedesis, phagocytosis, production and release of toxic effector molecules. The selectins and the leukocyte integrins of the CD18 family, including LFA-1 (CD11a/CD18), CR3 (CD11b/CD18), p150 (CD11c/CD18), are necessary for proper adhesion to endothelium. The complement opsonins C3b and C4b are recognized by CR1. IgG opsonins are recognized via the immunoglobulin receptors (FcγR). The stimulus dependent expression of these receptors is required for phagocytosis, degranulation and respiratory burst. The first microbicidal pathway is the oxidative reponse which consists of the production of ROI following NADPH-oxidase complex activation, including superoxide anion ($O_2^-$), hydrogen peroxide ($H_2O_2$) and via myeloperoxidase, hypochlorous acid (HOCl) and chloramines. The second microbicidal pathway is non-oxygen dependent and consists in the release in the phagolysosome or in the extracellular medium, of preformed proteins stored in granules. The serprocidins (serine proteases with antibiotic activity including elastase, cathepsin G, proteinase 3 and azurocidin) as well as myeloperoxidase are contained in the azurophilic granules. The metalloproteases (collagenase and gelatinase) are contained in specific and tertiary granules (for gelatinase only).

namely cytochrome b-245, or in cytosolic factors, phagocytes of CGD patients fail to mount a respiratory burst. Although their phagocytic capacity is normal, CGD phagocytes are incapable of producing ROI and of subsequently killing ingested pathogens.[9,10]

Neutrophils contain several thousand cytoplasmic granules which act as storage compartments for macromolecules destined for secretion, stored in specific granules, or for fusion with phagosomes, stored in azurophilic granules.[11–13] Neutrophils must degrade the connective tissues to extravasate and migrate to the site of inflammation. For this, they contain metalloproteinases such as collagenase, stored in the specific granules and gelatinase, stored in tertiary granules. The azurophilic granules contain the majority of the antibiotic proteins. Among the ten identified so far, two are thought to be unique in primary structure—lysosyme and bactericidal/permeability increasing protein (BPI).[14,15] The remaining eight fall into two families, each with four members: the defensins[16] on the one hand, and the serprocidins (serine proteinases with microbicidal activity) including elastase, cathepsin G, proteinase 3 and their enzymatically inactive homologue azurocidin, on the other.[17]

In neutrophils, ROI have indeed traditionally been viewed primarily as potent microbicidal agents. We would like to focus on the two paradigms that (1) despite the clearcut distinction between oxygen-dependent or non-dependent toxic mechanisms, the neutrophil is constructed to use both the NADPH oxidase system and the granule constituents in a cooperative and concerted manner and to realize its ultimate destructive potential; and (2) besides their toxic effects, ROI and proteases at lower concentrations may cooperate with other immunomodulatory molecules such as cytokines and adhesion molecules, and thus function as intercellular signalling molecules and to regulate phagocyte functions and modulate the overall inflammatory process.

*Neutrophil-derived reactive oxygen intermediates:*

*Biochemical basis of ROI-mediated effects.* An extensive report on mechanisms of cell injury by ROI will not be given; the reader can easily find several reviews on this subject in the literature.[18,19] Nevertheless, a brief outline of ROI biochemistry will be presented. There are three intermediates in the reduction of $O_2$ to $H_2O_2$. As a result, $O_2^-$, $H_2O_2$ and the hydroxyl radical (OH°) are formed by successive one electron additions. Despite numerous studies, the formation of OH° in phagocytes is still controversial.[20–23] So is the formation of singlet oxygen.[24,25] However, in this review, the term ROI will include $O_2^-$, $H_2O_2$, OH°, singlet oxygen and chlorinated oxidants such as HOCl and chloramines, and the defined oxygen species will be specified when possible.

Although the hallmark of the phagocyte remains the major source of ROI following NADPH oxidase activation, other cells can generate ROI through different pathways. Perhaps the most significant source of non-leukocyte generated ROI is the production of superoxide anion by xanthine oxidase, observed in ischaemia/reperfusion injury.[26,27] The cellular alterations following hypoxia promote the conversion of xanthine dehydrogenase to xanthine oxidase. Many other potential mechanisms derived from normal cellular metabolism could result in ROI generation, including mitochondrial transport, peroxisomal reactions and arachidonate metabolism.[28]

Free radicals and related oxidants have long been studied as agents of tissue damage. Lipid peroxidation is a well documented free radical chain reaction which can be initiated by OH° or transition metal complexes and remains an archetype of ROI-mediated toxicity.[29–31] Yet few studies have focused on its involvement in neutrophil oxidative reactions. Likewise, numerous studies have characterized the mechanisms underlying oxidative damage to RNA and DNA.[32–34]

In contrast, the reactions of proteins with various radicals/oxidants have not been so extensively studied although it is now clear that amino acids, peptides and proteins are, indeed, vulnerable to attack by a variety of ROI. Oxidation of particularly sensitive amino acid residues, aggregation or cross-linking, fragmentation and loss of enzymatic or other functional properties are but a few of the documented examples.[35–41]

Sulfhydryl groups can play an essential role in the function of several proteins, such as catalytic activity of enzymes and receptor binding capacity. These sulfhydryl groups are especially vulnerable to oxidative attack, with conversion to S–S bonds, particularly in the presence of metal ions. ROI, and particularly HOCl and chloramines, are all able to oxidize sulfhydryl groups resulting in the formation of a disulfide bridge. This oxidation could be corrected by thiol-containing molecules such as glutathione (GSH).[42,43]

Ultimately, oxidatively damaged proteins which exhibit higher proteolytic susceptibilities than other damaged proteins serve no useful role, and will be removed by specific proteolytic degradation. It is now clear that proteasome, a 670 kDa ATP-independent proteinase complex (also called macroxyproteinase) is responsible for most of the selective degradation of oxidatively modified proteins. This enzymatic complex exhibits a serine proteinase activity, a sulfhydryl proteinase activity and a metallo-peptidase activity.[44–46] The primary, secondary or tertiary structure of proteins may be altered depending on the oxidant and the overall result may be denaturation and increased hydrophobicity.[47]

Besides drastic oxidative attack on a protein, ROI may affect protein function by the oxidation of a critical residue in its active site with minor modification in the secondary or tertiary structure of the protein. This type of reaction could be defined more as 'oxidative regulation'[48] than 'oxidative attack'.

Many proteins may be oxidatively inactivated because of the sensitivity of methionine residues to oxidation. Much evidence suggests that methionine oxidation and subsequent loss of protein activity not only occur widely in living systems but are physiologic, homeostatic processes.[49] In some cases, methionine oxidation may occur without changes in physical or immunochemical properties and without loss of biologic activity, as is the case for the bovine growth hormone and the human α2-plasmin inhibitor.[50,51] Among the oxidants produced by activated neutrophils following the respiratory burst, $H_2O_2$ and the MPO-derived oxidants, namely HOCl and chloramines appear to be of biological relevance. By virtue of its strong oxidant potential, HOCl seems to be the toxic species used by neutrophils to mediate cell injury. Chloramines (*N*-chloroamine) arise from the chlorination of a wide spectrum of amine-containing compounds by HOCl.[6,52] Although these species differ in their reactivity with susceptible target molecules, they are able to mediate diverse biologic effects, ranging from the modulation of cellular functions to the induction of cell lysis. Chloramines have a half-life greater than radical oxygen species, ranging from 5 h at 37°C to more than 100 h at 4°C, depending on the surrounding environmental concentration of anti-oxidant. As a result, they can persist at sites of inflammation long after the disappearance of other oxygen metabolites, and have been termed long-lived oxidants. Generated chloramines are a mixture of chlorinated endogenous and exogenous amines of molecular weight ranging from 150 to 50 000.[53] The presence of exogenous amine-containing compounds can influence both the quantity and the characteristics of the long-lived oxidants generated. For example, the plasma protein albumin can be chlorinated and retain long-lived oxidant activity. The presence of appropriate nitrogenous compounds in the extracellular medium can result in the generation of lipophilic N–Cl oxidants of a strong cytotoxic potential. However, only hydrophilic chloramines are detected in supernatants from stimulated cells. Of primary physiologic importance among the hydrophilic generated chloramines is *N*-chlorotaurine formed by chlorination of the sulfur-containing amino acid taurine, which is plentiful in neutrophils.[54] Taurine is the major amine released into the supernatants of triggered neutrophils,[52] and thus its chloramine derivative is the most abundant.[53] Taurine chloramine is not a potent toxin but does oxidize proteins by specific attack at thioether moieties. The notion that methionine oxidation may be a relevant mechanism by which many proteins are regulated is corroborated by the fact that this oxidation can be reversed by methionine sulfoxide reductase, an enzyme which reduces methionine sulfoxide to methionine, using thioredoxin as cofactor.[55] The ability of this enzyme to reduce methionine sulfoxide in a variety of proteins suggests that it has broad specificity. It has been cloned and expressed in *Escherichia coli*.[56] Interestingly enough, it is widely distributed in different organs and in particular has been found at high concentration in neutrophil extracts. The ability of methionine sulfoxide reductase to reduce methionine sulfoxide residues suggests that it may be a repair enzyme for proteins inactivated by oxidation, or may participate in a novel type of post-translational regulation of proteins involved in the inflammatory process.

*Antioxidant-mediated cell protection*. Several antioxidant systems or ROI scavengers can protect tissue from oxidative damage.[57] Antioxidant defences appear to be an elaborate arsenal of antioxidants which function in concert to scavenge and detoxify ROI (superoxide dismutase (SOD), catalase, glutathione peroxidase), block free radical chain reactions (tocopherol, carotenoids and ascorbic acid), or sequester transition metals which can serve as a ready source of free electrons (lactoferrin, ceruloplasmin and transferrin).[58]

Owing to the chemical nature of ROI, especially radical species, it was difficult to measure these species directly. Thus, besides their physiologic role, ROI scavengers provide useful pharmacologic tools to probe cellular reactions in order to demonstrate the involvement of ROI in the underlying mechanisms.

Oxidants have regulatory effects by modulating antioxidant defences. Glutathione (GSH), the major non-protein thiol, is present in virtually all cell types and is involved in numerous biological functions.[59] It can act as a co-enzyme, an antioxidant by preserving intracellular reducing conditions, a regulatory molecule in cell cycle initiation and progression[60] and in microtubule formation.[61]

Regulation of intracellular content of GSH in lymphocytes has been shown to modify immune response capacity. The sensitivity of lymphocytes to oxidants is well established. For example, $H_2O_2$ impairs the proliferative capacity of human blood lymphocytes.[62,63] Several lymphocyte functions, such as mitogen-induced proliferation, natural killer activity, generation of immunoglobulins, are sensitive to the myeloperoxidase–$H_2O_2$—chloride system.[64,65] Thiol compounds, including GSH, are of critical significance.[66] The proliferative response is directly related to GSH availability.[67] Neutrophil derived chloramines markedly inhibit lymphocyte mitogen-induced proliferative response by decreasing GSH content.[68] GSH is involved in cytokine metabolism. It regulates interleukin-2 and interleukin-4 activity on cytotoxic T cells.[69,70] Interestingly, in an *in vitro* model of chronic HIV infection, GSH inhibits the induction of HIV expression suggesting that antioxidant therapy may be effective in limiting progression of the disease process.[71]

The importance of oxidant-mediated injury has prompted research on new antioxidant therapy by gene therapy. *In vitro* evaluation of the regulation of catalase gene expression in human airway epithelial cells has shown that the expression of the human catalase gene is not upregulated in these cells in response to hyperoxia, because the 5′-flanking region of the gene has characteristics of a non-regulated 'housekeeping' gene and does not respond to a hyperoxic stimulus. However, when human airway epithelial cells are infected with an adenoviral vector containing human catalase cDNA, catalase activity increases, as does the survival of cells subjected to oxidant stress.[72] A similar antioxidant strategy is efficient in protecting endothelial cells against $H_2O_2$-mediated injury.[73]

*Neutrophil-derived metalloproteinases and serine proteinases:* Neutrophil-derived proteinases are packed in granules which are released upon cell activation. Granule biogenesis follows the granulocyte differentiation pathway. The azurophilic granules first emerge at the stage of promyelocytes and contain neutral serine proteinases. Later in differentiation, at the stage of metamyelocytes, specific granules containing collagenase and gelatinase emerge.[74,75] However, a tertiary granule population containing gelatinase has been identified. The mechanisms underlying the secretion of the three morphologically distinct populations of granules may be under separate control. The order of exocytosis observed after ionophore-induced elevation of cytosolic calcium was gelatinase granules, specific granules, and lastly azurophilic granules.[76]

*Metalloproteinases*. Matrix metalloproteinases (MMPs) consitute a family of closely related enzymes which play important roles in a variety of physiological and pathological processes of matrix degradation. Human neutrophils contain both a collagenase and a gelatinase. One of the most intriguing aspects of the physicochemical properties of these neutrophil metalloproteinase is the fact that the enzyme can be isolated from the cell in a latent, inactive form. Neutrophil collagenase has been isolated as a 91 kDa latent enzyme that could be activated to yield active collagenase of 64 kDa which can degrade native interstitial collagens. Neutrophil gelatinase has also been isolated in its latent form, with reported molecular weights of 92, 130 and 225 kDa. The molecular basis underlying the latency of gelatinase has not been studied as extensively as that for collagenase. Collagenase attacks native interstitial collagen whereas gelatinase only degrades denatured collagen as well as native type IV or type V collagen.[77] Human neutrophil collagenase and gelatinase have been cloned.[78,79] Comparison of the primary structures of MMPs shows that they are structurally homologous with defined functional domains.[80,81] All these enzymes contain an essential catalytic zinc-binding domain, an $NH_2$-terminal domain which preserves the latent state of the enzyme and a COOH-terminal domain which plays a major role in substrate specificity. In the case of neutrophil collagenase, mutagenesis analysis has shown that substrate specificity is determined by a 16 amino-acid sequence in the COOH-terminal domain and is influenced by the integrity of a disulfide defined loop at the COOH-terminal for maximal activity. In addition, mutation of a critical aspartic residue at position 253 within the presumptive zinc-binding locus resulted in complete loss of proteolytic activity, suggesting that this aspartic residue might function as one of the ligands for divalent cations, which are essential for enzymatic activity.[82]

*Metalloproteinase inhibitors*. Secretion of metalloproteinase in an inactive precursor form is an important feature which regulates their activity in extracellular milieu. Organomercurials activate the proenzyme *in vitro* by inducing a conformational change. This reaction removes the amino terminal pro-segment, permanently converting the enzyme to the active form. Other mechanisms of activation may involve oxidants or serine proteinase. Further regulation of the activity of metalloproteinases in the extracellular milieu is achieved by specific inhibitors interacting with the activated enzymes. Metalloproteinases can be inhibited by α2-macroglobulin, a 725 kDa plasma proteinase inhibitor whose inhibitory properties are not specific and markedly different from all other known inhibitors.[77] Two tissue inhibitors of metalloproteinases (TIMP) have been characterized and cloned. TIMP-1 is a glycoprotein present in many tissues and biological fluids. It is secreted by several mammalian cell types, including fibroblasts, endothelial cells, smooth muscle cells and chondrocytes.[83–85] The isolation and cloning of a second metalloproteinase inhibitor, also called MI or TIMP-2, raises the question of the existence of a family of TIMP-like proteins. TIMP-2 displays 40% amino acid sequence homology with TIMP-1, with conservation of all twelve cysteine residues.[86–88] Both TIMP interact only with the activated enzyme and it has been shown that TIMP-2 has another regulation role which involves blocking the autoproteolytic activation of procollagenase.[89] The activity of TIMP is inhibited by different serine proteinases, including human neutrophil elastase, trypsin and α-chymotrypsin.[90] In addition, it seems that TIMP can be oxidatively regulated.

*Serine proteinases*. Serine proteinases are a large family of enzymes whose active site comprises the so-called 'catalytic triad' of histidine, aspartic acid and serine, in which a charge relay system allows the histidine and the aspartic acid to transiently bind a proton from the serine which can then attack the peptide bond in the target protein. In the azurophilic granules of the neutrophil, the group of the neutral serine proteinase homologues includes cathepsin G, elastase, proteinase 3 and azurocidin. Because they also possess microbicidal activity distinct from their proteolytic capacity, they will be referred to as serprocidins. Members of this family are cationic glycoproteins of similar size (25–29 kDa). They have all been cloned.[94–97] The serprocidins exhibit sequence homology between each other and with T cell proteinases, human lymphocyte proteinase, granzyme B, and rat mast cell proteinase.[13,95,98] Cloning of the genomic segment that contains the functional genes for neutrophil elastase, proteinase 3 (PR3), also called p29b or myeloblastin, and azurocidin, also called CAP37, has revealed that these genes form a cluster of genes, located in the terminal region of the short arm of chromosome 19 and are coordinately down-regulated in the promonocytic cell line U937 during induced terminal differentiation.[99] The gene of cathepsin G belongs to another cluster of genes encoding haematopoietic serine proteinases together with granzyme H and granzyme B genes on chromosome 14q11.2.[100,101]

Two well studied serprocidins include neutrophil elastase and cathepsin G. The remaining two, PR3 and azurocidin, more recently isolated and cloned, were identified as a result of a general screening for neutrophil-derived antibiotic proteins.[102,103] Neutrophil elastase and PR3 display very similar patterns of proteolytic activities.[104] They are both capable of cleaving insoluble elastin and a variety of matrix proteins, including fibronectin, laminin, vitronectin and collagen type IV. They show minimal activity against interstitial collagens, type I and III.[105,106] As a result, neutrophil elastase and PR3 have been shown to induce experimental pulmonary emphysema.[107–109]

Although the azurocidin sequence reveals extensive homology with serine proteinase, and in particular with neutrophil elastase, two amino acid substitutions, His→Ser and Ser→Gly, in the catalytic triad nullify its enzymatic activity.[95]

Cathepsin G shows little enzymatic activity compared with elastase. Nonetheless, specific substrates have been used to probe its proteolytic activity. Thanks to their proteolytic activity, serprocidins have been shown to be involved in platelet aggregation. Purified cathepsin G activates platelets in terms of aggregation, serotonin release, calcium movements and thromboxane $B_2$ formation.[110,111] Although neutrophil elastase or PR3 alone is unable to trigger platelet activation, each enhances cathepsin G induced platelet activation when added in combination with cathepsin G.[112]

*Serine proteinase inhibitors.* Neutrophil elastase, cathepsin G and PR3 are typical serine proteinases, which are rapidly inactivated by di-isopropyl fluorophosphate, synthetic acylating inhibitors[113] and specific serine proteinase inhibitors. The use of inhibitors seems to be the only way to regulate their activity inasmuch as these proteinases are probably functional in their packaged forms. Most likely the dipeptide activation fragment acts to process the enzyme for localization in the azurophilic granules; indeed, the nature of the enzyme responsible for the proteolytic cleavage is unknown.[114] Several human proteins function as potent inhibitors of elastase. Each has a characteristic physiological location, likely equivalent to its principal site of inhibitory action. The host's primary defence against uncontrolled elastase-mediated damage is α1-proteinase inhibitor (α1-PI, formerly termed α1-antitrypsin). Synthesized by hepatocytes, α1-PI is a 52 kDa glycoprotein, present at highest concentrations in plasma, but also found in human azurophilic granules.[115] Its high plasma concentration accounts for more than 90% of the elastase inhibitory capacity of human plasma.[116] α1-PI belongs to the serpin family composed of structurally homologous serine proteinase inhibitors. Interestingly, this typical tertiary structure is found in ovalbumin, one of the several members of the family with no known inhibitory function.[117] Members of this family have been identified in plants, insects and throughout the animal kingdom.[118] Most serpins are found in extracellular fluids but cytosolic serpins have been found in a wide variety of mammalian PMNs.[119,120]

When serpins interact with their cognate enzymes, a covalently stabilized serpin–enzyme complex is formed. The interaction results in a change in the conformation of the inhibitor. It has been shown that a pentapeptide domain in the carboxy-terminal fragment of α1-antitrypsin, which is highly conserved among the serpin family, is recognized by a specific cell surface receptor, the serpin enzyme complex (SEC), and through this SEC receptor is linked to several important physiological processes well characterized in the case of α1-PI, such as upregulation of the synthesis of α1-PI itself, internalization and endosomal/lysosomal degradation of SEC.[121–123]

Current concepts on the pathogenesis of emphysema largely emphasize the role of unrestrained proteolytic activity in the lung extracellular matrix. Since α1-PI provides almost all the protective screen of the lower respiratory tract against neutrophil elastase, emphysema might result from inactive α1-PI unable to inhibit neutrophil elastase in the lung.[124,125] Of particular interest is α1-PI deficiency, an autosomal hereditary disorder characterized by reduced levels of α1-PI in plasma and lung fluids, thereby leading to unopposed proteinase activity and culminating in pulmonary emphysema. The deficiency of α1-PI results from various mutations in five α1-PI coding exons.[126] Moreover, this absence of a normal anti-neutrophil elastase screen permits free elastase to bind to alveolar macrophages,[127,128] resulting in the release of leukotriene $B_4$ ($LTB_4$), a process which attracts neutrophils to the alveoli of α1-PI-deficient individuals, thus accelerating the lung destruction that characterizes this disorder.[129] Several strategies of α1-PI augmentation therapy for α1-PI deficiency can be used in order to restore a normal level of α1-PI.[130] An original approach consists in using endothelial cells as target for gene therapy of α1-PI deficiency, since the modified endothelial cell would secrete human α1-PI directly to the circulation, where it would diffuse into the alveolar interstitium, providing protection against neutrophil elastase. A modified adenovirus has been shown to transfer α1-PI cDNA into human endothelial cells, thus conferring upon the cells the ability to synthesize and secrete α1-PI capable of combining with neutrophil elastase.[131]

As for metalloproteinases, α2-macroglobulin inhibits elastase activity.[116] Another cell protein that inhibits leukocyte elastase is the secretory leukoproteinase inhibitor (SLPI), a 12 kDa non-glycosylated protein which is produced by cells of mucosal surfaces and found in the corresponding epithelial fluids, including in the lung. The molar concentrations of SLPI in total respiratory tract epithelial lining fluid (ELF) are 56 ± 10% that of α1-PI, suggesting that SLPI may be important for local anti-elastase protection. However, despite its relatively high concentration, functional studies have shown that only one-third is operative. Moreover, *in vitro* studies showed that exposure to oxidants combined to elastase causes the molecule to be cleaved from a 12 to an 8 kDa inactive fragment. It is conceivable that SLPI plays a first-line role, particularly in the upper airway where its concentration is highest. SLPI might be useful therapeutically in helping to augment the anti-elastase defences of the lung.[132] In an experimental rat model of immune complex-induced alveolitis, recombinant SLPI intra-tracheally instilled provided significant protection against pulmonary damage.[133]

A small acid-stable polypeptide of 7 kDa, elafin, is found in skin and appears to be an elastase inhibitor.

Human monocyte/neutrophil elastase inhibitor, a 42 kDa glycoprotein, and member of the serpin family, has recently been isolated and cloned.[119,120,134] A study of the reactive centre residues indicates that this new serpin seems to inhibit more than one proteinase and is likely to be oxidation sensitive. The latter feature would limit the sphere of action of EI to the immediate vicinity of carrier cells, thus regulating the proteolytic action of the neutrophil itself.

The substrate specificities of PR3 being so close to those of elastase, the pattern of inhibition with serine proteinase inhibitors are almost the same. PR3 can be inhibited by α1-PI, α2-macroglobulin or elafin, but not by SLPI. In addition, PR3 is able to selectively degrade both native and oxidized SLPI.[135] Its sensitivity to monocyte/neutrophil EI is unknown.

Cathepsin G displays a different pattern of inhibition, inasmuch as the serpin which accounted for the greatest inhibition is α1-antichymotrypsin (α1-AC). Moreover, elastase–α1-PI and cathepsin G–α1-antichymotrypsin complexes are chemotactic for neutrophils, this effect being mediated by the SEC receptor.[136–138] As for oxidant scavengers, synthetic proteinase inhibitors of narrow specificity have been developed to probe the involvement of proteinases in biological processes.

*Microbicidal and antibiotic activities of serprocidins.* Among purified neutrophil-derived proteinases, those that so far appear to have significant antibacterial potential independent of their enzymatic action are cathepsin G[139,140] and PR3.[106]

The antimicrobial potential of the proteinases could be expressed through an indirect mechanism involving antibiotic peptides synthesized as proforms, such as defensin.[141,142]

It is unknown how these antibiotic proteins stored in the granules as inactive proproteins undergo proteolytic cleavage for conversion to active cytotoxins, or where and when in the course of granulopoiesis this happens. However proteinases could be good candidates able to process and activate these antibiotic proteins, thus revealing an important role for these enzymes in antimicrobial events.[13]

*Role of proteinases in healing and wound repair.* Phagocytes, predominantly neutrophils and macrophages, play a critical role in early control of wound repair.[143] The movement of these cells is directed by the numerous chemoattractant substances that result from degradation of bacteria and autologous proteins.[144,145] The predominant action of the neutrophil in the wound is to express degradative enzymes and to provide an antibiotic shield through production of ROI and the release of antibiotic peptides. In the skin, neutrophils are responsible for the release of a variant form of collagenase, gelatinase and elastase. Proteinases participate both in destruction of invading micro-organisms and in removal of cellular and matrix debris. In addition, at least at some inflammatory sites, neutrophils can release other soluble signals such as IL-8, IL-1 and TGF-beta.[146]

*Serprocidins as target antigens for antineutrophil cytoplasmic antibodies.* Serprocidins are now recognized to be target antigens for antineutrophil cytoplasmic antibodies (ANCA) found in sera of patients with vasculitis, glomerulonephritis or other systemic inflammatory syndromes. When observed by indirect immunofluorescence microscopy on alcohol fixed PMN, ANCA can be divided into a group displaying a cytoplasmic staining pattern (C-ANCA) and a second group displaying perinuclear staining (P-ANCA).[147] Investigations of antigen specificity have been aimed at identifying the proteins recognized by these ANCA.[148] It appears that the majority of C-ANCA react with PR3, although in a few cases C-ANCA could be directed against BPI, elastase[149] or cathepsin G.[150] The major target antigen of P-ANCA is myeloperoxidase.[151]

Despite difficulties in classifying vasculitic syndromes, the correlation between clinical expression of Wegener's granulomatosis and ANCA reactivity has now well established that PR3 is the target autoantigen.[152,153] The pathogenesis of this form of immune necrotizing vasculitis that involves neither antibody directed against basement membrane nor immune complex deposition is only beginning to be understood.[154] Whether ANCA are serologic epiphenomena or play a pathogenic role in the course of the disease is still a matter of debate.

Many studies have focused on *in vitro* ANCA-induced activation of neutrophils and consequent damage to endothelial cells. It is possible that ANCA target antigens such as PR3 translocate to the cell surface to bind ANCA and then trigger the respiratory burst and subsequent release of ROI.[155,156] Other studies have been directed at characterization of these autoantigens, thus pointing out that there are different species of C-ANCA that bind different epitopes of PR3 thus increasing the difficulty of assessing the pathophysiological meaning of these ANCAs.[157,158]

### ROI and proteinases in the modulation of inflammation

*Cooperation between ROI and proteinases in host tissue damage:* Modulation of the proteolytic capacity of neutrophils by their chlorinated oxidants relies on their ability to inactivate serine proteinase inhibitors by oxidation as well as to activate latent metalloproteinases such as collagenase or gelatinase, thus potentiating the resulting deleterious effect of the proteinases.[159]

The α1-PI contains a methionine residue critical to its activity at position 358[160] which can be oxidized and thus inactivated by hypochlorous acid or chloramines from activated neutrophils. Oxidation of Met-358 causes a 2 000-fold decrease in the rate of association between neutrophil elastase and the modified antiproteinase. Recombinant α1-PI resistant to oxidation does not undergo inactivation and efficiently protects connective tissue from neutrophil damage.[161,162] Some nonsteroidal anti-inflammatory drugs appear to rescue α1-PI from oxidative inactivation by efficiently limiting the extracellular availability of HOCl in the neutrophil surroundings.[163] Inasmuch as both α2-macroglobulin and SLPI appear to be sensitive to oxidation, the complete anti-elastase defence is regulated by neutrophil-derived chlorinated oxidants. The direct effect of oxidant attack on elastase using free radicals produced in a Fenton reaction ($H_2O_2$ in the presence of copper) shows that elastase, as well as three of its inhibitors, eglin c, α1-PI and SLPI, are efficiently inactivated.[164] Study of the effect of chloramines on α-chymotrypsin, another serine proteinase, has shown that oxidation barely modifies its catalytic properties, whereas sensitivity to specific proteinase inhibitors is decreased.[165] Study of the synergy between myeloperoxidase-$H_2O_2$-chloride system and elastase in degrading endothelial cell matrix heparan sulfate proteoglycan has shown that elastase alone, and the myeloperoxidase system alone, cause degradation, and when a 4 h exposure to elastase was followed by 15 min of the myeloperoxidase system, the effect was greater than additive. No such effect was seen when both systems were added together, or when elastase followed the myeloperoxidase system.[166] Studies of the interactions of elastase with its insoluble substrate, elastin, have shown that elastase forms a stable complex with elastin-derived peptides during elastinolysis and, as a result, elastin derived peptides could contribute to modulation of the proteolytic activity of elastase.[167]

Metalloproteinases are stored in latent form within granules. When neutrophils are stimulated to release their lysosomal enzymes, these latent enzymes must be activated before they can attack their substrate. The mechanisms by which neutrophil-derived oxidants activate latent metalloproteinases is still not completely understood, but latent collagenase can be activated by HOCl whereas progelatinase seems to require both oxidant- and serine proteinase-dependent pathways.[168–170] Some reports indicate that cathepsin G is a key mediator in neutrophil collagenase activation, and that HOCl under certain conditions leads to activation of collagenase or the stimulation of cathepsin G ability to activate neutrophil collagenase.[171] Moreover, the activity of metalloproteinases is also regulated via their specific inhibitors, the TIMPs, which can be inactivated by oxidation or proteolytic cleavage.[90,172] The modulatory effect of ROI on neutrophil-derived serine proteinases and matrix metalloproteinases appears to be of crucial importance in the inflammatory lung process where these enzymes play a key role in acute lung damage. The co-administration of the serine proteinase inhibitor SLPI and the metalloproteinase inhibitor TIMP significantly prevents pulmonary damage in a rat model of immune complex-induced alveolitis.[133] The inactivation of human α1-PI can occur by interaction with bacterial proteinases such as seryl–cysteinyl– and metalloproteinases from *Staphylococcus aureus*. This process of inactivation of α1-PI by pathogenic proteinases could amplify dysregulation of elastase activity during septicaemia.[173]

Of special relevance in the study of proteinases/antiproteinases and oxidant/antioxidant balances is the case of cystic fibrosis. Cystic fibrosis is a hereditary disorder caused by mutations of the cystic fibrosis transmembrane conductance regulator (CFTR) the product of which is a membrane protein thought to function as a chloride channel. The lethal clinical manifestations are clearly related to the thick, infected mucus and chronic neutrophil-dominated airway inflammation.[174–176] In these patients, chronic airway infection, especially by *Pseudomonas aeruginosa*, is of critical important in prognosis. This pathogen can never be permanently eradicated despite intensive antibiotic treatment and leads invariably to respiratory failure, which is the cause of death in most patients with cystic fibrosis. It is now well established that neutrophil-derived oxidants inactivate antiproteinases such as α1-PI, thus potentiating the deleterious effects of elastase. Moreover, antiproteinase imbalance has been recognized as a factor favouring the persistence of *Pseudomonas aeruginosa*.[177–179] In order to increase local antiproteinase defence, different aerosol treatments have been tested in cystic fibrosis patients. Aerosol administration of α1-PI efficiently suppresses the burden of active elastase on the respiratory epithelial surface.[180] Likewise aerosolized recombinant SLPI reduces elastase levels and lung inflammatory state.[181]

*Interactions of ROI and proteinases with pro-inflammatory cytokines*: There is now evidence for several possible functional points of contact between ROI and proteinases, on the one hand, and cytokines, on the other. Cytokines can be regulated at different levels, e.g. the molecule itself, its inhibitor or its receptor, and at different stages such as transcription, synthesis or activity. An additional level of regulation is possible inasmuch as the effector mechanisms of cytokine activities could involve ROI interacting with transcription factors.

Given the wide diversity of cytokines involved in the control of the inflammatory process and the immune response,[182] we will focus only on tumour necrosis factor-α (TNFα) and the chemokine IL-8, both of which are able to activate neutrophils and trigger ROI generation. They will be used as representative examples in our discussion.

*Tumour necrosis factor-α*. TNFα is a cytokine which has important functions in coordinating immunological and inflammatory responses. TNFα is released primarily by activated macrophages and induces changes in cell shape, differentiation and proliferation of a variety of cell types, including T and B cells, neutrophils, macrophages, thymocytes, endothelial cells, keratinocytes, glial cells and fibroblasts.[183] The interaction between TNFα and ROI is two-way since TNFα induces neutrophils to produce ROI,[184–186] which in turn acts on TNFα expression and release. Lastly, ROI seem to be involved in the transduction pathway of TNFα as well as in its transcription control through the nuclear factor NF kappa B.[187]

Evidence suggests that ROI might provide a positive signal for the release of TNFα and, in various cells, TNFα-mediated cell lysis can be inhibited by thioredoxin[188] or *N*-acetylcysteine, a thiol antioxidant and GSH precursor.[189–191] TNFα induces oxidative stress in isolated mouse hepatocytes. Hepatocytes exposed to a TNFα concentration that does not induce cell toxicity exhibited intracellular GSH depletion and GSSG efflux during the first 2 h of exposure, followed by a decrease in cellular ATP content. The antioxidants mannitol and benzoate, as well as the iron chelator desferoxamine, reduce the extent of TNFα induced oxidative stress, suggesting involvement of the hydroxyl radical.[192] In mesangial cells, TNFα, as well as aggregated IgG, increase mRNA levels for the monocyte chemoattractant protein, JE/MCP-1, and the colony-stimulating factor, CSF-1. Superoxide anion generated by the xanthine oxidase system, but not $H_2O_2$ increases mRNA levels for both JE/MCP-1 and CSF-1. Addition of NADPH to activate membrane bound NADPH-oxidase dependent superoxide generating system increases mRNA levels and further enhances stimulation by TNFα. It appears that generation of ROI, possibly via a NADPH-oxidase pathway, is involved in the induction of JE/MCP-1 and CSF-1 genes by TNFα and aggregated IgG complexes.[193] The finding that pretreatment of sensitive targets with TNFα induces resistance to TNFα was attributed to the synthesis of the mitochondrial form of superoxide dismutase, MnSOD.[194] Moreover, cellular sensitivity and resistance to TNFα are correlated, respectively, with decreased and increased levels of superoxide dismutase. The effect of TNFα on the expression of antioxidant enzymes has been studied in TNFα sensitive myeloid cell lines and in normal peripheral blood lymphocytes and monocytes. Exposure to TNFα induces a striking increase in manganese superoxide dismutase (MnSOD) RNA levels whereas no changes are observed in the case of copper-zinc superoxide dismutase (Cu/ZnSOD) or glutathione peroxidase. Furthermore, TNFα resistant leukaemic cell lines have higher constitutive levels of MnSOD RNA and activity, and these levels do not change in the presence of TNFα.[195] The effects of IL-1, TNFα and LPS treatment on the expression of superoxide dismutases have been studied in both rat pulmonary artery and microvascular endothelial cells. Similar results were obtained insofar as these mediators produced an increase in MnSOD but not Cu/ZnSOD expression in both tested cell lines.[196] In contrast, another study showed that two cell lines in which mutants have been selected to be resistant to cytolysis by TNFα behave differently. The SK-MEL-109 variants had relatively low levels of MnSOD that were inducible by TNFα; the HeLa variants had very low levels of MnSOD that were poorly inducible by TNFα. It was thus concluded that an elevated level of MnSOD was not required to protect these cells from TNFα mediated cytolysis.[197]

Glutathione appears to be of critical importance in TNFα-mediated effects. In bovine pulmonary microvascular endothelial cells, stimulation with TNFα increases the permeability induced by $H_2O_2$, at a concentration which by itself has no effect on the permeability of endothelial cells. Pretreatment with TNFα for 6 h had no direct effect on transendothelial permeability. However, this TNFα pretreatment enhanced the susceptibility of endothelial cells to $H_2O_2$. Measurement of intracellular antioxidant content following exposure to TNFα revealed a decrease in reduced GSH and an increase in the oxidized form (GSSG), whereas no change in catalase content could be detected. The decrease in oxidant buffering capacity secondary to TNFα-induced reduction in intracellular GSH content mediates the increased susceptibility of endothelial cells to $H_2O_2$.[198] The same type of study has been performed with phorbol myristate acetate (PMA)-activated neutrophils as ROI generating system. Challenge of neutrophils layered on TNFα-pretreated endothelial cells with PMA increases permeability. In contrast, challenge of CGD-neutrophils fails to induce any change in endothelial cell permeability.[199]

It is still unclear whether ROI are implicated in TNFα-induced injury derived from the phagocyte respiratory burst or from the action of xanthine oxidase, and further studies are needed to address this issue. However, it is possible that intracellular ROI may result in the activation of genes responsible for apoptosis, conceivably through an oxidative stress-responsive nuclear transcription factor such as NF-kappa B.[200]

The regulatory effect of $H_2O_2$ on both the cytotoxic activity and the specific binding of TNFα was studied in TNFα-sensitized murine L929 cells. Half-hour pretreatment with $H_2O_2$ altered cell sensitivity to TNFα, whereas $H_2O_2$ preatreatment did not modify TNFα activity. The inhibitory effect of $H_2O_2$ was suppressed by catalase; but was unaffected by the scavengers of OH– and HOCl, suggesting that $H_2O_2$, and not one of its metabolites, was responsible for this inhibition. The $H_2O_2$ effect was associated with an approximately 50% decrease in density of cell membrane TNFα receptors, without any change in their affinity. Moreover $H_2O_2$ did not affect the rate of degradation of TNFα receptors. It appears that $H_2O_2$ can down-regulate the cellular response to TNFα, possibly by reducing its binding capacity.[201]

A growing body of evidence has emerged supporting the implication of proteinases in the modulation of cellular responses to cytokines,[202] and especially to TNFα. Specific proteolytic cleavage of the molecules themselves can lead to release of membrane anchored precursors, facilitate secretion and abolish or generate biological activities. As a result, proteolysis can affect specific cell surface receptors and free their extracellular domains to circulate as soluble inhibitors of cytokine activity.

Two types of TNFα receptors have been isolated in the human and their genes have been cloned and structure activity studies carried out.[203–206] The type I receptor is a 55 kDa molecule which is expressed preferentially in cells of epithelial origin, whereas the 75 kDa type II receptor is more abundant in myeloid cells, human neutrophils and monocytes, expressing similar amounts of each receptor at their surface. The mechanisms of TNFα receptor shedding are still not completely understood. The release of either type I or type II receptor seems to involve different proteolytic pathways. A first TNFα releasing activity has been localized in azurophilic granules and identified by its inhibitory pattern as elastase. This enzyme preferentially acts on the p75 TNFα receptor of neutrophils and mononuclear cells, releasing a soluble fragment of 32 kDa that retains its ability to bind TNFα. A second TNFα releasing activity insensitive to serine–proteinase inhibitor is operative in intact fMLP-stimulated neutrophils, shedding a 42 kDa fragment from the p75 TNFα receptor and a 28 kDa fragment from the p55 TNFα receptor.[207] It appears that the mechanisms underlying TNFα receptor shedding involve proteinases other than serine proteinases contained in azurophilic granules, and further studies are needed to localize and identify these proteinases.

The pathophysiological importance of the interactions between oxidants, proteinases and pro-inflammatory cytokines has been extensively studied by our own group in patients with end-stage renal disease receiving haemodialysis therapy.[208] It is now clear that each dialysis session triggers neutrophil activation, mainly through the generation of activated complement components following the contact of blood with bioincompatible dialysis membranes.[209] This is evidenced by massive generation of ROI[210] and the presence of high levels of neutrophil elastase and α1-PI.[211] This perdialytic activation contrasts with an overall depression in the expected responses of these cells to pathogens, e.g. chemotactic, phagocytic and oxidative response capacities.[212] Among the numerous side effects of ROI that could account for dialysis related complications, the possibility that they could contribute to β2 microglobulin amyloidosis arthropathy, by inducing fragmentation, polymerization and thus favouring subsequent intra-articular deposit of β2 microglobulin, has recently been raised by our study of the effect of defined radiolytically generated oxygen species on β2 microglobulin structure.[41]

Acting in concert with neutrophil-derived ROI and proteinases, monocyte-derived circulating pro-inflammatory cytokines could also play a critical role in dialysis related chronic inflammatory process and contribute to the underlying immune system dysregulation associated with uraemia.[213]

Recently, high levels of both TNF–sR55 and TNF–sR75 and an imbalance between TNFa and its soluble receptors, acting as naturally occurring inhibitors,[214] have been reported in dialysis patients.[215,216] It thus seems that as with the oxidant/antioxidant balance the cytokine/anti-cytokine balance could play a critical role in complications observed in long-term dialysis patients.

*Interleukin-8.* IL-8 belongs to a novel class of small cytokines, now called chemokines, which are widely studied because of their ability to activate leukocytes and their potential role as mediators of inflammation. IL-8 has been described as a potent neutrophil chemoattractant and activator.[217] Studies using whole blood as a model of cytokine production have shown that the $OH^-$ scavenging agent dimethyl sulfoxide (DMSO) drastically inhibits IL-8 production in blood stimulated with lipopolysaccharide (LPS) or other agents such as TNFα, IL-1β, phytohaemagglutinin A (PHA) and aggregated immune complexes.[218] However, using the same whole blood model, in both normal individuals and CGD patients, DMSO inhibitable production of IL-8 could be triggered in response to LPS, PHA or aggregated immune complexes. Direct exposure to $H_2O_2$ stimulates IL-8 production in a concentration-dependent manner in a hepatoma cell line, Hep-G2, a pulmonary type II epithelial cell line, A549, and human skin fibroblasts.[219] It seems that ROI are important regulators of IL-8 expression whether or not they are derived from NADPH oxidase activation.

Oxidative regulation of IL-8 activity could be mediated through its receptor. Studies in neutrophils have shown that two sulfhydryl groups participate in the binding of the ligand to the receptor and consequently regulate receptor-mediated cell functions.[220]

There is evidence that neutrophil-derived proteinases are involved in the control of IL-8 production. The fluid lining the respiratory epithelium in cystic fibrosis contains large numbers of neutrophils and, consequently, active neutrophil elastase. Neutrophil elastase appears to be the mediator responsible for inducing bronchial epithelial cells to express IL-8 gene and release neutrophil chemotactic activity. In addition, neutrophil elastase inhibitors prevent IL-8 gene expression.[221,222] Aerosolization of recombinant SLPI in patients with cystic fibrosis suppresses the neutrophil elastase burden on the respiratory epithelial surface and at the same time greatly reduces IL-8 levels, thus decreasing the number of PMN in the epithelial lining fluid.[181] The role of IL-8 in lung inflammatory reactions has been demonstrated by the protection afforded by treatment with an anti-IL-8 monoclonal antibody in a model of E-selectin-dependent lung injury.[223]

Studies of the modulation of IL-8 production have revealed the critical role of ROI or proteinases alone or in combination. These new findings could form the basis of new therapeutic practice in the management of inflammation.

*Interactions of ROI and proteinases with adhesion molecules:* The interaction of neutrophils with endothelial cells, one of the first events in the acute inflammatory response, induces profound changes in the biosynthesis of potent endothelial modulators. Although it has been known for more than a decade that endothelial cells can be damaged *in vitro* by neutrophil derived ROI,[224,225] it is now apparent that sub-injurious concentrations of ROI could alter the physiologic status of endothelial cells and modify their response to inflammatory mediators.

Mechanisms of endothelial cell killing by neutrophil-derived ROI can be blocked by superoxide dismutase, catalase, iron chelators or scavengers of the hydroxyl radical. There is evidence that products from xanthine oxidase of endothelial cells are necessary for the toxic effects of hydrogen peroxide or phorbol ester-activated neutrophils. Conversion of xanthine dehydrogenase to xanthine oxidase in endothelial cells occurs during contact of endothelial cells with neutrophils. This conversion is not related to neutrophil-derived ROI.[226] The glutathione redox cycle has been shown to protect cultured endothelial cells against lysis by extracellularly generated $H_2O_2$[227] and appears to be critical for the survival of tumour cells exposed to $H_2O_2$.[228]

Adhesion of circulating PMN to endothelial cells is a critical step in the inflammatory process. This complex cell/cell interaction requires increased expression of surface adhesive molecules either on neutrophils or on endothelial cells. The adherence of human neutrophils to human endothelial cells *in vitro* is increased by chemotactic stimulation of the neutrophils,[229,230] or by stimulation of endothelial cells with endotoxin[231] or cytokines such as TNFα or IL-1β.[232–235] It has been recognized that within the post-capillary venules of inflamed tissue, emigrating leukocytes leave the central stream of the circulation, roll more slowly along the vessel wall, then stop by adhering tightly to the endothelial surface before squeezing between adjacent endothelial cells into the sub-endothelial tissue.[236] More recently, it has been appreciated that the rolling phenomenon is mediated by the selectin class of adhesion molecules, while the leukocyte β2 integrins (CD11/CD18) and their endothelial counterreceptors, (intercellular adhesion molecule or ICAM) are involved in the tight adhesion.[237–239] CD11/CD18 receptors on neutrophils are known to modulate chemotaxis, neutrophil/neutrophil aggregation, neutrophil adhesion and transvascular migration.[240] In addition, other adhesion molecules are involved in transendothelial migration of leukocytes such as the platelet/endothelial cell adhesion molecule 1 (PECAM-1).[241,242] Recent evidence indicates that ROI and proteinases modulate adhesion molecule functions. In a model of cat mesenteric venule, $H_2O_2$ and chloramines but not HOCl, promote leukocyte adhesion to venular endothelium. $H_2O_2$-induced CD18-mediated leukocyte adherence appears to be elicited by PAF and by a direct effect of the oxidant on CD11/CD18 expression.

Treatment with low concentrations of $H_2O_2$ (50 to 100 μM) selectively increases the surface expression and mRNA levels of ICAM-1 and major histocompatibility complex class I, but not endothelial E-selectin, vascular cell adhesion molecule-1 (VCAM-1), or gp96, a constitutively expressed endothelial cell surface protein. $H_2O_2$ does not activate the transcription nuclear factor kappa B, an important mediator of TNFα-induced gene expression. It seems that subinjurious doses of $H_2O_2$ can activate endothelium, the molecular mechanisms underlying this phenomenon differing from those of inflammatory cytokines.[243] After exposure to the hypoxanthine/xanthine oxidase ROI producing system, human umbilical vein endothelial cells display increased adherence to resting neutrophils, compared with untreated endothelial cells. ICAM-1, constitutively present on the surface of resting endothelial cells, is involved in neutrophil adherence to ROI treated cells, since this adherence is inhibited by anti-ICAM1, anti-CD11a, anti-CD11b and anti-CD18 monoclonal antibodies.[244] Another study showed that endothelial cells exposed to $H_2O_2$ express the granule membrane protein-40 (GMP-40), which can be translocated from its intracellular storage pool to the surface of endothelial cells where it acts as a ligand for neutrophil adhesion.[245] Similar results were obtained in an *in vitro* model of hypoxia/reoxygenation induced endothelial free-radical generation and adhesion of resting PMN. Oxidative stress induced an increase in the expression of GMP-40 as well as *de novo* synthesis of endothelial adhesion molecule 1 (ELAM-1) on the endothelial surface, these effects being blocked by free radical scavengers.[246]

One possible interaction between proteinases and membrane proteins such as adhesion molecules, is proteolytic cleavage, leading to soluble factors, as has been described for TNFα receptors. It is well documented that the circulating neutrophils respond to inflammatory products by expressing surface receptors appropriate to the inflammatory signal.[247] Typically, this expression involves exposure or functional activation of opsonin receptors including receptors for Fc fragment of IgG (FcγR) and receptors for complement components. Neutrophils possess two classes of FcγR which have different functions, as well as different sensitivity to proteolytic cleavage by neutrophil elastase. FcγRII (CD32) is made up of an external opsonin-binding portion, a membrane domain and a cytoplasmic tail, whereas FcγRIII (CD16) is anchored to the phagocyte membrane through a phosphatidylinositol linkage. FcγRII, which is resistant to elastase, is primarily responsible for IgG mediated activation of human PMN since FcγRIII, which is sensitive to elastase, binds immune complexes without directly activating neutrophils.[248]

Complement receptors include the opsonin receptor CR1 (CD35) which binds C3b and C4b, the chemotactic receptor for C5a and CR3, a member of the integrin family (CD11b/CD18 or Mac-1) which recognizes C3bi and ICAM as its endothelial counterpart receptor. Expression of both CR1 and CR3 is increased upon neutrophil activation[249] and in the presence of cytokines.[250]

Interestingly enough an upregulation of CR3 on both monocytes and neutrophils has been reported in dialysis patients during the session[251] providing a molecular mechanism for dialysis induced neutrophil sequestration in the pulmonary vascular bed.

The expression of both CR1 and CR3 has also been studied in circulating or bronchoalveolar lavage (BAL) neutrophils in the case of cystic fibrosis patients compared with controls. In circulating neutrophils, CR1 and CR3 expression was similar both in cystic fibrosis patients and controls. In BAL, CR3 expression was also similar to the controls, whereas CR1 expression was significantly decreased and neutrophil elastase was, at least in part, responsible for the proteolytic cleavage of CR1.[252]

Leukosialin is the major sialoprotein of human leukocytes.[253,254] Due to the net negative charge of leukosialin, its function may be to prevent nonspecific cell/cell interactions through negative charge repulsions. Phorbol myristate acetate (PMA) or fMLP activation of neutrophils reduces the expression of leukosialin via a shedding mechanism, blocked by both metalloproteinase and serine proteinase inhibitors.[255–257] Nonetheless, both exogenous and endogenous elastase have been reported to be responsible for this shedding. Moreover, in participating in the shedding of such a strongly anionic membrane protein which drastically modifies the neutrophil surface charge, neutrophil-derived proteinases participate in the regulation of the cell adhesion process.

In order to assess the *in vivo* role of elastase in neutrophil adhesion and emigration, intravital microscopy has been used to study PMN/endothelial cell interactions in single inflamed post-capillary venules in a model of platelet activating factor (PAF) superfusion onto the mesentery. *In vivo*, elastase significantly contributes to neutrophil infiltration, aggregation, adhesion and emigration, these effects being blocked by elastase inhibitors. Moreover, the direct action of elastase on neutrophils assessed *in vitro* indicates that neutrophil elastase directly upregulates the β subunit of the CD11/CD18 glycoprotein complex without any change in L-selectin levels. Moreover, the increase in CD18 expression appears to be independent of degranulation, superoxide anion production and cell lysis. By selectively enhancing and regulating CD18 dependent chemotaxis, a rate-limiting step for other neutrophil functions *in vivo*,[258] elastase may modulate neutrophil infiltration and consequently the overall migration process.[259]

### Conclusions

A combination of reductionist and integrationist approaches is rapidly expanding and revising our understanding of the role of phagocyte-derived ROI and proteinases. For nearly two decades, investigation of the toxic potential of ROI prompted numerous studies of different cell types aimed at describing and characterizing the mechanisms of cell lysis, the target cell involved and the defences used to counteract ROI. Because of the new concept that ROI could have a role as signalling molecules, investigation has focused on the effect of sub-lethal and non-injurious concentrations of ROI on known biological functions of given cell types. Studies of the involvement of ROI and proteinases in different regulatory processes suggest an overall system of cooperation. A growing body of evidence supports this notion of cooperation in the field of cytokines, as illustrated by TNFα and IL-8, and in the field of adhesion molecules, both fields having several points in common with ROI and proteinases.

The neutrophil itself appears to be well equipped with this unusual combination of contrasting molecules: ROI, small, ubiquitous and short-lived molecules; and proteinases, macromolecules with specific long-lasting activity. The neutrophil safely keeps them apart through separate intracellular compartmentalization under basal conditions. Nevertheless, in the defence of the host against pathogens, their cooperation maximizes neutrophil microbicidal potential as well as damage to host tissue. However, the cooperation appears to be involved at another level, where they act more as immunomodulatory mediators than toxic weapons, in the whole inflammatory process. The overall picture is of a classical alliance, for better and for worse.

### References

1. Fauve RM. Inflammation and natural immunity. In: Fougereau M, Dausset J, eds. *Progress in Immunology*. London: Academic Press, 1980; 737–756.
2. Weiss SJ. Tissue destruction by neutrophils. *N Engl J Med* 1989; **320**: 365–376.
3. Klebanoff SJ. Oxygen metabolites from phagocytes. In: Gallin JI, Goldstein IM, Snyderman R, eds. *Inflammation: Basic Principles and Clinical Correlates*. New York: Raven Press, 1992; 541–589.
4. Babior BM. The respiratory burst of phagocytes. *J Clin Invest* 1984; **73**: 599–601.
5. Nathan CF. Neutrophil activation on biological surfaces. Massive secretion of hydrogen peroxide in response to products of macrophages and lymphocytes. *J Clin Invest* 1987; **80**: 1550–1560.
6. Test ST, Weiss SJ. The generation and utilization of chlorinated oxidants by human neutrophils. *Adv Free Rad Biol Med* 1986; **2**: 91–116.
7. Winterbourn CC. Neutrophil oxidants: production and reactions. In: Das DK, Essman WB, eds. *Oxygen Radicals: Systemic Events and Disease Process*. Basel: Karger, 1990; 31–70.
8. Rotrosen D. The respiratory burst oxidase. In: Gallin JI, Goldstein IM, Snyderman R, eds. *Inflammation: Basic Principles and Clinical Correlates*. New York: Raven Press, 1992; 589–601.
9. Gallin JI, Leto TL, Rotrosen D, Kwong CH, Malech HL. Delineation of the phagocyte NADPH oxidase through studies of chronic granulomatous diseases of childhood. *Curr Opin Immunol* 1991; **4**: 53–56.
10. Segal AW, Abo A. The biochemical basis of the NADPH oxidase of phagocytes. *Trends Biochem Sci* 1993; **18**: 43–47.
11. Spitznagel JK. Antibiotic proteins of human neutrophils. *J Clin Invest* 1990; **86**: 1381–1386.
12. Lehrer RI, Ganz T. Antimicrobial polypeptides of human neutrophils. *Blood* 1990; **76**: 2169–2181.
13. Elsbach P, Weiss J. Oxygen-independent antimicrobial systems of phagocytes. In: Gallin JI, Goldstein IM, Snyderman R, eds. *Inflammation: Basic Principles and Clinical Correlates*. New York: Raven Press, 1992; 603–636.
14. Elsbach P, Weiss J. The bactericidal/permeability-increasing protein (BPI), a potent element in host-defense against gram-negative bacteria and lipopolysaccharide. *Immunobiology* 1993; **187**: 417–429.
15. Elsbach P, Weiss J. Bactericidal/permeability increasing protein and host defense against gram-negative bacteria and endotoxin. *Curr Opin Immunol* 1993; **5**: 103–107.
16. Ganz T, Selsted ME, Lehrer RI. Defensins. *Eur J Haematol* 1990; **44**: 1–8.
17. Gabay JE, Almeida RP. Antibiotic peptides and serine protease homologs in human polymorphonuclear leukocytes: defensins and azurocidin. *Curr Opin Immunol* 1993; **5**: 97–102.
18. Halliwell B. Oxidants and human disease: some new concepts. *FASEB J* 1987; **1**: 358–364.
19. Farber JL, Kyle ME, Coleman JB. Mechanisms of cell injury by activated oxygen species. *Lab Invest* 1990; **62**: 670–679.
20. Tauber AI, Babior BM. Evidence for hydroxyl radical production by human neutrophils. *J Clin Invest* 1977; **60**: 374–379.
21. Rosen H, Klebanoff SJ. Hydroxyl radical generation by polymorphonuclear leukocytes measured by electron spin resonance spectroscopy. *J Clin Invest* 1979; **64**: 1725–1729.
22. Britigan BE, Rosen GM, Chai Y, Cohen MS. Do human neutrophils make hydroxyl radical? Determination of free radicals generated by human neutrophils activated with a soluble or particulate stimulus using electron paramagnetic resonance spectrometry. *J Biol Chem* 1986; **261**: 4426–4431.
23. Ward PA, Till GO, Kunkel R, Beauchamp C. Evidence for role of hydroxyl radical in complement and neutrophil-dependent tissue injury. *J Clin Invest* 1983; **72**: 789–801.
24. Allen RC, Stjernholm RL, Steele RH. Evidence for the generation of an electronic excitation state(s) in human polymorphonuclear leukocytes and its participation in bactericidal activity. *Biochem Biophys Res Commun* 1972; **47**: 679–684.
25. Harrison JE, Watson BD, Schultz J. Myeloperoxidase and singlet oxygen: a reappraisal. *FEBS Lett* 1978; **92**: 327–331.
26. Granger DN. Role of xanthine oxidase and granulocytes in ischemia-reperfusion injury. *Am J Physiol* 1988; **255**: H1269–H1275.
27. McCord JM. Oxygen-derived radicals: a link between reperfusion injury and inflammation. *Fed Proc* 1987; **46**: 2402–2406.
28. Freeman BA, Crapo JD. Free radicals and tissue injury. *Lab Invest* 1982; **47**: 412–426.
29. Slater TF. Free-radical mechanisms in tissue injury. *Biochem J* 1984; 222: 1–15.
30. McCord JM. Oxygen-derived free radicals in postischemic tissue injury. *N Engl J Med* 1985; **312**: 159–163.
31. Halliwell B, Gutteridge JM. Oxygen toxicity, oxygen radicals, transition metals and disease. *Biochem J* 1984; **219**: 1–14.
32. Imlay JA, Linn S. DNA damage and oxygen radical toxicity. *Science* 1988; **240**: 1302–1309.
33. Dizdaroglu M. Chemical determination of free radical-induced damage to DNA. *Free Radic Biol Med* 1991; **10**: 225–242.
34. Joenje H. Genetic toxicology of oxygen. *Mutat Res* 1989; **219**: 193–208.
35. Davies KJ. Protein damage and degradation by oxygen radicals. I. general aspects. *J Biol Chem* 1987; **262**: 9895–9901.
36. Wolff SP, Garner A, Dean RT. Free radicals, lipids and protein degradation. *Trends Biochem Sci* 1986; **11**: 27–31.
37. Salo DC, Lin SW, Pacifici RE, Davies KJ. Superoxide dismutase is preferentially degraded by a proteolytic system from red blood cells following oxidative modification by hydrogen peroxide. *Free Radic Biol Med* 1988; **5**: 335–339.
38. Vissers MC, Winterbourn CC. Oxidative damage to fibronectin. I. The effects of the neutrophil myeloperoxidase system and HOCl. *Arch Biochem Biophys* 1991; **285**: 53–59.
39. Franzini E, Sellak H, Hakim J, Pasquier C. Oxidative damage to lysozyme by the hydroxyl radical: comparative effects of scavengers. *Biochim Biophys Acta* 1993; **1203**: 11–17.
40. Monboisse JC, Braquet P, Randoux A, Borel JP. Non-enzymatic degradation of acid-soluble calf skin collagen by superoxide ion: protective effect of flavonoids. *Biochem Pharmacol* 1983; **32**: 53–58.
41. Capeillere-Blandin C, Delaveau T, Descamps-Latscha B. Structural modifications of human beta 2 microglobulin treated with oxygen-derived radicals. *Biochem J* 1991; **277**: 175–182.
42. Silverstein RM, Hager LP. The chloroperoxidase-catalyzed oxidation of thiols and disulfides to sulfenyl chlorides. *Biochemistry* 1974; **13**: 5069–5073.
43. Armstrong DA, Buchanan JD. Reactions of $O_2^-$, $H_2O_2$ and other oxidants with sulfhydryl enzymes. *Photochem Photobiol* 1978; **28**: 743–755.
44. Pacifici RE, Salo DC, Davies KJ. Macroxyproteinase (M.O.P.): a 670 kDa proteinase complex that degrades oxidatively denatured proteins in red blood cells. *Free Radic Biol Med* 1989; **7**: 521–536.
45. Davies KJ, Lin SW, Pacifici RE. Protein damage and degradation by oxygen radicals. IV. Degradation of denatured protein. *J Biol Chem* 1987; **262**: 9914–9920.
46. Davies KJ. Intracellular proteolytic systems may function as secondary antioxidant defenses: an hypothesis. *J Free Radic Biol Med* 1986; **2**: 155–173.
47. Pacifici RE, Kono Y, Davies KJ. Hydrophobicity as the signal for selective degradation of hydroxyl radical-modified hemoglobin by the multicatalytic proteinase complex, proteasome. *J Biol Chem* 1993; **268**: 15405–15411.
48. Ossanna PJ, Test ST, Matheson NR, Regiani S, Weiss SJ. Oxidative regulation of neutrophil elastase-alpha-1-proteinase inhibitor interactions. *J Clin Invest* 1986; **77**: 1939–1951.
49. Swaim MW, Pizzo SV. Methionine sulfoxide and the oxidative regulation of plasma proteinase inhibitors. *J Leukoc Biol* 1988; **43**: 365–379.
50. Glaser CB, Li CH. Reaction of bovine growth hormone with hydrogen peroxide. *Biochemistry* 1974; **13**: 1044–1047.
51. Shieh BH, Travis J. The reactive site of human alpha 2-antiplasmin. *J Biol Chem* 1987; **262**: 6055–6059.
52. Grisham MB, Jefferson MM, Melton DF, Thomas EL. Chlorination of endogenous amines by isolated neutrophils. Ammonia-dependent bactericidal, cytotoxic, and cytolytic activities of the chloramines. *J Biol Chem* 1984; **259**: 10404–10413.

53. Test ST, Lampert MB, Ossanna PJ, Thoene JG, Weiss SJ. Generation of nitrogen–chlorine oxidants by human phagocytes. *J Clin Invest* 1984; **74**: 1341–1349.
54. Aruoma OI, Halliwell B, Hoey BM, Butler J. The antioxidant action of taurine, hypotaurine and their metabolic precursors. *Biochem J* 1988; **256**: 251–255.
55. Brot N, Weissbach L, Werth J, Weissbach H. Enzymatic reduction of protein-bound methionine sulfoxide. *Proc Natl Acad Sci USA* 1981; **78**: 2155–2158.
56. Rahman MA, Nelson H, Weissbach H, Brot N. Cloning, sequencing, and expression of the *Escherichia coli* peptide methionine sulfoxide reductase gene. *J Biol Chem* 1992; **267**: 15549–15551.
57. Heffner JE, Repine JE. Pulmonary strategies of antioxidant defense. *Am Rev Respir Dis* 1989; **140**: 531–554.
58. Halliwell B. Drug antioxidant effects. A basis for drug selection? *Drugs* 1991; 42: 569–605.
59. Meister A, Anderson ME. Glutathione. *Annu Rev Biochem* 1983; **52**: 711–760.
60. Messina JP, Lawrence DA. Cell cycle progression of glutathione-depleted human peripheral blood mononuclear cells is inhibited at S phase. *J Immunol* 1989; **143**: 1974–1981.
61. Burchill BR, Olivier JM, Pearson CB, Leinbach ED, Berlin RD. Microtubule dynamics and gluthatione metabolism in phagocytozing human polymorphonuclear leukocytes. *J Cell Biol* 1978; **76**: 439–447.
62. Sagone AL Jr, Husney R, Guter H, Clark L. Effect of catalase on the proliferation of human lymphocytes to phorbol myristate acetate. *J Immunol* 1984; **133**: 1488–1494.
63. Farber CM, Liebes LF, Kanganis DN, Silber R. Human B lymphocytes show greater susceptibility to $H_2O_2$ toxicity than T lymphocytes. *J Immunol* 1984; **132**: 2543–2546.
64. El-Hag A, Lipsky PE, Bennett M, Clark RA. Immunomodulation by neutrophil myeloperoxidase and hydrogen peroxide: differential susceptibility of human lymphocyte functions. *J Immunol* 1986; **136**: 3420–3426.
65. Staite ND, Messner RP, Zoschke DC. Inhibition of human T lymphocyte E rosette formation by neutrophils and hydrogen peroxide. Differential sensitivity between helper and suppressor T lymphocytes. *J Immunol* 1987; **139**: 2424–2430.
66. Gougerot-Pocidalo MA, Fay M, Roche Y, Chollet-Martin S. Mechanisms by which oxidative injury inhibits the proliferative response of human lymphocytes to PHA. Effect of the thiol compound 2-mercaptoethanol. *Immunology* 1988; **64**: 281–288.
67. Hamilos DL, Zelarney P, Mascali JJ. Lymphocyte proliferation in glutathione-depleted lymphocytes: direct relationship between glutathione availability and the proliferative response. *Immunopharmacology* 1989; **18**: 223–235.
68. Witko-Sarsat V, Nguyen AT, Descamps-Latscha B. Immunomodulatory role of phagocyte-derived chloramines involving lymphocyte glutathione. *Mediators Inflam* 1993; **2**: 235–241.
69. Liang CM, Lee N, Cattell D, Liang SM. Glutathione regulates interleukin-2 activity on cytotoxic T-cells. *J Biol Chem* 1989; **264**: 13519–13523.
70. Liang SM, Lee N, Finbloom DS, Liang CM. Regulation by glutathione of interleukin-4 activity on cytotoxic T cells. *Immunology* 1992; **75**: 435–440.
71. Kalebic T, Kinter A, Poli G, Anderson ME, Meister A, Fauci AS. Suppression of human immunodeficiency virus expression in chronically infected monocytic cells by glutathione, glutathione ester, and *N*-acetylcysteine. *Proc Natl Acad Sci USA* 1991; **88**: 986–990.
72. Yoo JH, Erzurum SC, Hay JG, Lemarchand P, Crystal RG. Vulnerability of the human airway epithelium to hyperoxia. Constitutive expression of the catalase gene in human bronchial epithelial cells despite oxidant stress. *J Clin Invest* 1994; **93**: 297–302.
73. Erzurum SC, Lemarchand P, Rosenfeld MA, Yoo JH, Crystal RG. Protection of human endothelial cells from oxidant injury by adenovirus-mediated transfer of the human catalase cDNA. *Nucleic Acids Res* 1993; **21**: 1607–1612.
74. Fouret P, Du Bois RM, Bernaudin JF, Takahashi H, Ferrans VJ, Crystal RG. Expression of the neutrophil elastase gene during human bone marrow cell differentiation. *J Exp Med* 1989; **169**: 833–845.
75. Henson PM, Henson JE, Fittschen C, Bratton DL, Riches DWH. Degranulation and secretion by phagocytic cells. In: Gallin JI, Goldstein IM, Snyderman R, eds. *Inflammation: Basic Principles and Clinical Correlates.* New York: Raven Press, 1992; 511–539.
76. Sengelov H, Kjeldsen L, Borregaard N. Control of exocytosis in early neutrophil activation. *J Immunol* 1993; **150**: 1535–1543.
77. Weiss SJ, Peppin GJ. Collagenolytic metalloenzymes of the human neutrophil. *Biochem Pharmacol* 1986; **35**: 3189–3197.
78. Hasty KA, Tayebeh F, Pourmotabbed T., *et al.* Human neutrophil collagenase. A distinct gene product with homology to other matrix metalloproteinases. *J Biol Chem* 1990; **265**: 11421–11424.
79. Devarajan P, Johnston JJ, Ginsberg SS, Van Wart HE, Berliner N. Structure and expression of neutrophil gelatinase cDNA. Identity with type IV collagenase from HT1080 cells. *J Biol Chem* 1992; **267**: 25228–25232.
80. Wilhelm SM, Collier IE, Marmer BL, Eisen AZ, Grant GA, Goldberg GI. SV40-transformed human lung fibroblasts secrete a 92-kDa type IV collagenase which is identical to that secreted by normal human macrophages. *J Biol Chem* 1989; **264**: 17213–17221.
81. Sanchez-Lopez R, Nicholson R, Gesnel MC, Matrisian LM, Breathnach R. Structure–function relationships in the collagenase family member transin. *J Biol Chem* 1988; **263**: 11892–11899.
82. Hirose T, Patterson C, Pourmotabbed T, Mainardi CL, Hasty KA. Structure–function relationship of human neutrophil collagenase: identification of regions responsible for substrate specificity and general proteinase activity. *Proc Natl Acad Sci USA* 1993; **90**: 2569–2573.
83. Cawston TE, Galloway WA, Mercer E, Murphy G, Reynolds JJ. Purification of rabbit bone inhibitor of collagenase. *Biochem J* 1981; **195**: 159–165.
84. Stricklin GP, Welgus HG. Human skin fibroblast collagenase inhibitor. Purification and biochemical characterization. *J Biol Chem* 1983; **258**: 12252–12258.
85. DeClerck YA, Yean TD, Chan D, Shimada H, Langley KE. Inhibition of tumor invasion of smooth muscle cell layers by recombinant human metalloproteinase inhibitor. *Cancer Res* 1991; **51**: 2151–2157.
86. Boone TC, Johnson MJ, DeClerck YA, Langley KE. cDNA cloning and expression of a metalloproteinase inhibitor related to tissue inhibitor of metalloproteinases. *Proc Natl Acad Sci USA* 1990; **87**: 2800–2804.
87. Stetler-Stevenson WG, Krutzsch HC, Liotta LA. Tissue inhibitor of metalloproteinase (TIMP-2). A new member of the metalloproteinase inhibitor family. *J Biol Chem* 1989; **264**: 17374–17378.
88. Goldberg GI, Marmer BL, Grant GA, Eisen AZ, Wilhelm S, He CS. Human 72-kilodalton type IV collagenase forms a complex with a tissue inhibitor of metalloproteases designated TIMP-2. *Proc Natl Acad Sci USA* 1989; **86**: 8207–8211.
89. Declerck YA, Yean TD, Lu HS, Ting J, Langley KE. Inhibition of autoproteolytic activation of interstitial procollagenase by recombinant metalloproteinase inhibitor MI/TIMP-2. *J Biol Chem* 1991; **266**: 3893–3899.
90. Okada Y, Watanabe S, Nakanishi I, *et al.* Inactivation of tissue inhibitor of metalloproteinases by neutrophil elastase and other serine proteinases. *FEBS Lett* 1988; **229**: 157–160.
91. Salvesen G, Farley D, Shuman J, Przybyla A, Reilly C, Travis J. Molecular cloning of human cathepsin G: structural similarity to mast cell and cytotoxic T lymphocyte proteinases. *Biochemistry* 1987; **26**: 2289–2293.
92. Takahashi H, Nukiwa T, Yoshimura K, *et al.* Structure of the human neutrophil elastase gene. *J Biol Chem* 1988; **263**: 14739–14747.
93. Bories D, Raynal MC, Solomon DH, Darzynkiewicz Z, Cayre YE. Down-regulation of a serine protease, myeloblastin, causes growth arrest and differentiation of promyelocytic leukemia cells. *Cells* 1989; **59**: 959–968.
94. Campanelli D, Melchior M, Fu Y, *et al.* Cloning of cDNA for proteinase 3: a serine protease, antibiotic, and autoantigen from human neutrophils. *J Exp Med* 1990; **172**: 1709–1715.
95. Almeida RP, Melchior M, Campanelli D, Nathan C, Gabay JE. Complementary DNA sequence of human neutrophil azurocidin, an antibiotic with extensive homology to serine proteases. *Biochem Biophys Res Commun* 1991; **177**: 688–695.
96. Morgan JG, Sukiennicki T, Pereira HA, Spitznagel JK, Guerra ME, Larrick JW. Cloning of the cDNA for the serine protease homolog CAP37/azurocidin, a microbicidal and chemotactic protein from human granulocytes. *J Immunol* 1991; **147**: 3210–3214.
97. Sturrock AB, Franklin KF, Rao G, *et al.* Structure, chromosomal assignment, and expression of the gene for proteinase-3. The Wegener's granulomatosis autoantigen. *J Biol Chem* 1992; **267**: 21193–21199.
98. Hudig D, Ewoldt GR, Woodard SL. Proteases and lymphocyte cytotoxic killing mechanisms. *Curr Opin Immunol* 1993; **5**: 90–96.
99. Zimmer M, Medcalf RL, Fink TM, Mattmann C, Lichter P, Jenne DE. Three human elastase-like genes coordinately expressed in the myelomonocyte lineage are organized as a single genetic locus on 19pter. *Proc Natl Acad Sci USA* 1992; **89**: 8215–8219.
100. Hanson RD, Hohn PA, Popescu NC, Ley TJ. A cluster of hematopoietic serine protease genes is found on the same chromosomal band as the human alpha/delta T-cell receptor locus. *Proc Natl Acad Sci USA* 1990; **87**: 960–963.
101. Haddad P, Jenne D, Tschopp J, Clement MV, Mathieu-Mahul D, Sasportes M. Structure and evolutionary origin of the human granzyme H gene. *Int Immunol* 1991; **3**: 57–66.
102. Gabay JE, Heiple JM, Cohn ZA, Nathan CF. Subcellular location and properties of bactericidal factors from human neutrophils. *J Exp Med* 1986; **164**: 1407–1421.
103. Gabay JE, Scott RW, Campanelli D, *et al.* Antibiotic proteins of human polymorphonuclear leukocytes. *Proc Natl Acad Sci USA* 1989; **86**: 5610–5614.
104. Kam CM, Kerrigan JE, Dolman KM, Goldschmeding R, Von Dem Borne AE, Powers JC. Substrate and inhibitor studies on proteinase 3. *FEBS Lett* 1992; **297**: 119–123.
105. Rao NV, Wehner NG, Marshall BC, Gray WR, Gray BH, Hoidal JR. Characterization of proteinase-3 (PR-3), a neutrophil serine proteinase. Structural and functional properties. *J Biol Chem* 1991; **266**: 9540–9548.
106. Campanelli D, Detmers PA, Nathan CF, Gabay JE. Azurocidin and a homologous serine protease from neutrophils. Differential antimicrobial and proteolytic properties. *J Clin Invest* 1990; **85**: 904–915.
107. Janoff A, Sloan B, Weinbaum G, *et al.* Experimental emphysema induced with purified human neutrophil elastase: tissue localization of the instilled protease. *Am Rev Respir Dis* 1977; **115**: 461–478.
108. Senior RM, Tegner H, Kuhn C, Ohlsson K, Starcher BC, Pierce JA. The induction of pulmonary emphysema with human leukocyte elastase. *Am Rev Respir Dis* 1977; **116**: 469–475.
109. Kao RC, Wehner NG, Skubitz KM, Gray BH, Hoidal JR. Proteinase 3. A distinct human polymorphonuclear leukocyte proteinase that produces emphysema in hamsters. *J Clin Invest* 1988; **82**: 1963–1973.
110. Selak MA, Chignard M, Smith JB. Cathepsin G is a strong platelet agonist released by neutrophils. *Biochem J* 1988; **251**: 293–299.
111. Renesto P, Ferrer-Lopez P, Chignard M. Interference of recombinant eglin C, a proteinase inhibitor extracted from leeches, with neutrophil-mediated platelet activation. *Lab Invest* 1990; **62**: 409–416.
112. Renesto P, Halbwachs-Mecarelli L, Nusbaum P, Lesavre P, Chignard M. Proteinase 3: a neutrophil proteinase with an activity on platelets. *J Immunol* 1994 (in press).
113. Powers JC, Carroll DL. Reaction of acyl carbazates with proteolytic enzymes. *Biochem Biophys Res Commun* 1975; **67**: 639–644.
114. Watorek W, Farley D, Salvesen G, Travis J. Neutrophil elastase and cathepsin G: structure, function, and biological control. *Adv Exp Med Biol* 1988; **240**: 23–31.

115. Mason DY, Cramer EM, Masse JM, Crystal R, Bassot JM, Breton-Gorius J. Alpha 1-antitrypsin is present within the primary granules of human polymorphonuclear leukocytes. *Am J Pathol* 1991; **139**: 623–628.
116. Travis J, Salvesen GS. Human plasma proteinase inhibitors. *Annu Rev Biochem* 1983; **52**: 655–709.
117. Stein PE, Leslie AG, Finch JT, Turnell WG, McLaughlin PJ, Carrell RW. Crystal structure of ovalbumin as a model for the reactive centre of serpins. *Nature* 1990; **347**: 99–102.
118. Kanost MR, Prasad SV, Wells MA. Primary structure of a member of the serpin superfamily of proteinase inhibitors from an insect, *Manduca sexta*. *J Biol Chem* 1989; **264**: 965–972.
119. Remold-O'Donnell E, Nixon JC, Rose RM. Elastase inhibitor. Characterization of the human elastase inhibitor molecule associated with monocytes, macrophages, and neutrophils. *J Exp Med* 1989; **169**: 1071–1086.
120. Thomas RM, Nauseef WM, Iyer SS, Peterson MW, Stone PJ, Clark RA. A cytosolic inhibitor of human neutrophil elastase and cathepsin G. *J Leukoc Biol* 1991; **50**: 568–579.
121. Perlmutter DH, Glover GI, Rivetna M, Schasteen CS, Fallon RJ. Identification of a serpin-enzyme complex receptor on human hepatoma cells and human monocytes. *Proc Natl Acad Sci USA* 1990; **87**: 3753–3757.
122. Joslin G, Fallon RJ, Bullock J, Adams SP, Perlmutter DH. The SEC receptor recognizes a pentapeptide neodomain of alpha 1-antitrypsin-protease complexes. *J Biol Chem* 1991; **266**: 11282–11288.
123. Perlmutter DH, Joslin G, Nelson P, Schasteen C, Adams SP, Fallon RJ. Endocytosis and degradation of alpha 1-antitrypsin-protease complexes is mediated by the serpin–enzyme complex (SEC) receptor. *J Biol Chem* 1990; **265**: 16713–16716.
124. Stockley RA. Alpha-1-antitrypsin and the pathogenesis of emphysema. *Lung* 1987; **165**: 61–77.
125. Janoff A. Elastases and emphysema. Current assessment of the protease–antiprotease hypothesis. *Am Rev Respir Dis* 1985; **132**: 417–433.
126. Crystal RG, Brantly ML, Hubbard RC, Curiel DT, States DJ, Holmes MD. The alpha 1-antitrypsin gene and its mutations. Clinical consequences and strategies for therapy. *Chest* 1989; **95**: 196–208.
127. Campbell EJ, White RR, Senior RM, Rodriguez RJ, Kuhn C. Receptor-mediated binding and internalization of leukocyte elastase by alveolar macrophages *in vitro*. *J Clin Invest* 1979; **64**: 824–833.
128. Campbell EJ. Human leukocyte elastase, cathepsin G, and lactoferrin: family of neutrophil granule glycoproteins that bind to an alveolar macrophage receptor. *Proc Natl Acad Sci USA* 1982; **79**: 6941–6945.
129. Hubbard RC, Fells G, Gadek J, Pacholok S, Humes J, Crystal RG. Neutrophil accumulation in the lung in alpha 1-antitrypsin deficiency. Spontaneous release of leukotriene $B_4$ by alveolar macrophages. *J Clin Invest* 1991; **88**: 891–897.
130. Hubbard RC, Crystal RG. Alpha-1-antitrypsin augmentation therapy for alpha-1-antitrypsin deficiency. *Am J Med* 1988; **84**: 52–62.
131. Lemarchand P, Jaffe HA, Danel C, *et al.* Adenovirus-mediated transfer of a recombinant human alpha 1-antitrypsin cDNA to human endothelial cells. *Proc Natl Acad Sci USA* 1992; **89**: 6482–6486.
132. Vogelmeier C, Hubbard RC, Fells GA, *et al.* Anti-neutrophil elastase defense of the normal human respiratory epithelial surface provided by the secretory leukoprotease inhibitor. *J Clin Invest* 1991; **87**: 482–488.
133. Mulligan MS, Desrochers PE, Chinnaiyan AM, *et al. In vivo* suppression of immune complex-induced alveolitis by secretory leukoproteinase inhibitor and tissue inhibitor of metalloproteinases 2. *Proc Natl Acad Sci USA* 1993; **90**: 11523–11527.
134. Remold-O'Donnell E, Chin J, Alberts M. Sequence and molecular characterization of human monocyte/neutrophil elastase inhibitor. *Proc Natl Acad Sci USA* 1992; **89**: 5635–5639.
135. Rao NV, Marshall BC, Gray BH, Hoidal JR. Interaction of secretory leukocyte protease inhibitor with proteinase-3. *Am J Respir Cell Mol Biol* 1993; **8**: 612–616.
136. Banda MJ, Rice AG, Griffin GL, Senior RM. Alpha 1-proteinase inhibitor is a neutrophil chemoattractant after proteolytic inactivation by macrophage elastase. *J Biol Chem* 1988; **263**: 4481–4484.
137. Banda MJ, Rice AG, Griffin GL, Senior RM. The inhibitory complex of human alpha 1-proteinase inhibitor and human leukocyte elastase is a neutrophil chemoattractant. *J Exp Med* 1988; **167**: 1608–1615.
138. Joslin G, Griffin GL, August AM, *et al.* The serpin–enzyme complex (SEC) receptor mediates the neutrophil chemotactic effect of alpha-1 antitrypsin-elastase complexes and amyloid-beta peptide. *J Clin Invest* 1992; **90**: 1150–1154.
139. Shafer WM, Pohl J, Onunka VC, Bangalore N, Travis J. Human lysosomal cathepsin G and granzyme B share a functionally conserved broad spectrum antibacterial peptide. *J Biol Chem* 1991; **266**: 112–116.
140. Bangalore N, Travis J, Onunka VC, Pohl J, Shafer WM. Identification of the primary antimicrobial domains in human neutrophil cathepsin G. *J Biol Chem* 1990; **265**: 13584–13588.
141. Daher KA, Lehrer RI, Ganz T, Kronenberg M. Isolation and characterization of human defensin cDNA clones. *Proc Natl Acad Sci USA* 1988; **85**: 7327–7331.
142. Ganz T, Rayner JR, Valore EV, Tumolo A, Talmadge K, Fuller F. The structure of the rabbit macrophage defensin genes and their organ-specific expression. *J Immunol* 1989; **143**: 1358–1365.
143. Davidson JM. Wound repair. In: Gallin JI, Goldstein IM, Snyderman R, eds. *Inflammation: Basic Principles and Clinical Correlates*. New York: Raven Press, 1992; 809–819.
144. Postlethwaite AE, Kang AH. Collagen- and collagen peptide-induced chemotaxis of human blood monocytes. *J Exp Med* 1976; **143**: 1299–1307.
145. Postlethwaite AE, Keski-Oja J, Balian G, Kang AH. Induction of fibroblast chemotaxis by fibronectin. Localization of the chemotactic region to a 140,000-molecular weight non-gelatin-binding fragment. *J Exp Med* 1981; **153**: 494–499.
146. Grotendorst GR, Smale G, Pencev D. Production of transforming growth factor beta by human peripheral blood monocytes and neutrophils. *J Cell Physiol* 1989; **140**: 396–402.
147. Wieslander J. How are antineutrophil cytoplasmic autoantibodies detected? *Am J Kidney Dis* 1991; **18**: 154–158.
148. Lesavre P. Antineutrophil cytoplasmic autoantibodies antigen specificity. *Am J Kidney Dis* 1991; **18**: 159–163.
149. Nassberger L, Jonsson H, Sjoholm AG, Sturfelt G, Heubner A. Circulating anti-elastase in systemic lupus erythematosus (letter). *Lancet* 1989; **i**: 509.
150. Halbwachs-Mecarelli L, Nusbaum P, Noel LH, *et al.* Antineutrophil cytoplasmic antibodies (ANCA) directed against cathepsin G in ulcerative colitis, Crohn's disease and primary sclerosing cholangitis. *Clin Exp Immunol* 1992; **90**: 79–84.
151. Falk RJ, Jennette JC. Anti-neutrophil cytoplasmic autoantibodies with specificity for myeloperoxidase in patients with systemic vasculitis and idiopathic necrotizing and crescentic glomerulonephritis. *N Engl J Med* 1988; **318**: 1651–1657.
152. Jenne DE, Tschopp J, Ludemann J, Utecht B, Gross WL. Wegener's autoantigen decoded. *Nature* 1990; **346**: 520.
153. Ewert BH, Jennette JC, Falk RJ. The pathogenic role of antineutrophil cytoplasmic autoantibodies. *Am J Kidney Dis* 1991; **18**: 188–195.
154. Hagen EC, Ballieux BE, Van Es LA, Daha MR, Van Der Woude FJ. Antineutrophil cytoplasmic autoantibodies: a review of the antigens involved, the assays, and the clinical and possible pathogenetic consequences. *Blood* 1993; **81**: 1996–2002.
155. Falk RJ, Terrell RS, Charles LA, Jennette JC. Anti-neutrophil cytoplasmic autoantibodies induce neutrophils to degranulate and produce oxygen radicals *in vitro*. *Proc Natl Acad Sci USA* 1990; **87**: 4115–4119.
156. Keogan MT, Esnault VL, Green AJ, Lockwood CM, Brown DL. Activation of normal neutrophils by anti-neutrophil cytoplasm antibodies. *Clin Exp Immunol* 1992; **90**: 228–234.
157. Van de Wiel BA, Dolman KM, Van Der Meer-Gerritsen CH, Hack CE, Von Dem Borne AE, Goldschmeding R. Interference of Wegener's granulomatosis autoantibodies with neutrophil proteinase 3 activity. *Clin Exp Immunol* 1992; **90**: 409–414.
158. Bini P, Gabay JE, Teitel A, Melchior M, Zhou JL, Elkon KB. Antineutrophil cytoplasmic autoantibodies in Wegener's granulomatosis recognize conformational epitope(s) on proteinase 3. *J Immunol* 1992; **149**: 1409–1415.
159. Weiss SJ, Regiani S. Neutrophils degrade subendothelial matrices in the presence of alpha-1-proteinase inhibitor. Cooperative use of lysosomal proteinases and oxygen metabolites. *J Clin Invest* 1984; **73**: 1297–1303.
160. Johnson D, Travis J. The oxidative inactivation of human alpha-1-proteinase inhibitor. Further evidence for methionine at the reactive center. *J Biol Chem* 1979; **254**: 4022–4026.
161. George PM, Vissers MC, Travis J, Winterbourn CC, Carrell RW. A genetically engineered mutant of alpha 1-antitrypsin protects connective tissue from neutrophil damage and may be useful in lung disease. *Lancet* 1984; **ii**: 1426–1428.
162. Steffens GJ, Heinzel-Wieland R, Saunders D, *et al.* Oxidation resistant muteins of antileukoproteinase as potential therapeutic agents. *Agents Actions Suppl* 1993; **42**: 111–121.
163. Dallegri F, Ottonello L, Dapino P, Bevilacqua M. The anti-inflammatory drug nimesulide rescues alpha-1-proteinase inhibitor from oxidative inactivation by phagocytosing neutrophils. *Respiration* 1992; **59**: 1–4.
164. Dean RT, Nick HP, Schnebli HP. Free radicals inactivate human neutrophil elastase and its inhibitors with comparable efficiency. *Biochem Biophys Res Commun* 1989; **159**: 821–827.
165. Cutruzzola F, Ascenzi P, Barra D, *et al.* Selective oxidation of Met-192 in bovine alpha-chymotrypsin. Effect on catalytic and inhibitor binding properties. *Biochim Biophys Acta* 1993; **1161**: 201–208.
166. Klebanoff SJ, Kinsella MG, Wight TN. Degradation of endothelial cell matrix heparan sulfate proteoglycan by elastase and the myeloperoxidase–$H_2O_2$–chloride system. *Am J Pathol* 1993; **143**: 907–917.
167. Tyagi SC, Simon SR. Regulation of neutrophil elastase activity by elastin-derived peptide. *J Biol Chem* 1993; **268**: 16513–16518.
168. Weiss SJ, Peppin G, Ortiz X, Ragsdale C, Test ST. Oxidative autoactivation of latent collagenase by human neutrophils. *Science* 1985; **227**: 747–749.
169. Peppin GJ, Weiss SJ. Activation of the endogenous metalloproteinase, gelatinase, by triggered human neutrophils. *Proc Natl Acad Sci USA* 1986; **83**: 4322–4326.
170. Vissers MC, Winterbourn CC. Activation of human neutrophil gelatinase by endogenous serine proteinases. *Biochem J* 1988; **249**: 327–331.
171. Capodici C, Muthukumaran G, Amoruso MA, Berg RA. Activation of neutrophil collagenase by cathepsin G. *Inflammation* 1989; **13**: 245–258.
172. Kramps JA, Van Twisk C, Klasen EC, Dijkman JH. Interactions among stimulated human polymorphonuclear leucocytes, released elastase and bronchial antileucoprotease. *Clin Sci* 1988; **75**: 53–62.
173. Potempa J, Watorek W, Travis J. The inactivation of human plasma alpha 1-proteinase inhibitor by proteinases from *Staphylococcus aureus*. *J Biol Chem* 1986; **261**: 14330–4334.
174. Welsh MJ, Fick RB. Cystic fibrosis. *J Clin Invest* 1987; **80**: 1523–1526.
175. McIntosh I, Cutting GR. Cystic fibrosis transmembrane conductance regulator and the etiology and pathogenesis of cystic fibrosis. *FASEB J* 1992; **6**: 2775–2782.
176. Jennings CA, Crystal RG. Inflammatory lung disease: molecular determinants of emphysema, bronchitis, and fibrosis. In: Gallin JI, Goldstein IM, Snyderman R, eds. *Inflammation: Basic Principles and Clinical Correlates*. New York: Raven Press, 1992; 983–997.
177. Doring G, Albus A, Hoiby N. Immunologic aspects of cystic fibrosis. *Chest* 1988; **94**: 109S–115S.
178. Suter S. Imbalance between polymorphonuclear leukocyte proteases and antiproteases in chronic pyogenic infections and its relation to the proteolysis of complement component C3. *Complement* 1986; **3**: 1–24.

179. Goldstein W, Döring G. Lysosomal enzymes from polymorphonuclear leukocytes and proteinase inhibitors in patients with cystic fibrosis. *Am Rev Respir Dis* 1986; **134**: 49–56.
180. McElvaney NG, Hubbard RC, Birrer P *et al.* Aerosol alpha 1-antitrypsin treatment for cystic fibrosis. *Lancet* 1991; **337**: 392–394.
181. McElvaney NG, Nakamura H, Birrer P *et al.* Modulation of airway inflammation in cystic fibrosis. *In vivo* suppression of interleukin-8 levels on the respiratory epithelial surface by aerosolization of recombinant secretory leukoprotease inhibitor. *J Clin Invest* 1992; **90**: 1296–1301.
182. Nathan C, Sporn M. Cytokines in context. *J Cell Biol* 1991; **113**: 981–986.
183. Beutler B, Cerami A. The biology of cachectin/TNFα a primary mediator of the host response. *Annu Rev Immunol* 1989; **7**: 625–655.
184. Nathan C, Srimal S, Farber C, *et al.* Cytokine-induced respiratory burst of human neutrophils: dependence on extracellular matrix proteins and CD11/CD18 integrins. *J Cell Biol* 1989; **109**: 1341–1349.
185. Laudanna C, Miron S, Berton G, Rossi F. Tumor necrosis factor-alpha/cachectin activates the O2(−)-generating system of human neutrophils independently of the hydrolysis of phosphoinositides and the release of arachidonic acid. *Biochem Biophys Res Commun* 1990; **166**: 308–315.
186. Fuortes M, Jin WW, Nathan C. Adhesion-dependent protein tyrosine phosphorylation in neutrophils treated with tumor necrosis factor. *J Cell Biol* 1993; **120**: 777–784.
187. Larrick JW, Wright SC. Cytotoxic mechanism of tumor necrosis factor-alpha. *FASEB J* 1990; **4**: 3215–3223.
188. Larrick JW, Wright SC. Cytotoxic mechanism of tumor necrosis factor-alpha. *FASEB J* 1990; **4**: 3215–3223.
189. Chang DJ, Ringold GM, Heller RA. Cell killing and induction of manganous superoxide dismutase by tumor necrosis factor-alpha is mediated by lipoxygenase metabolites of arachidonic acid. *Biochem Biophys Res Commun* 1992; **188**: 538–546.
190. Droge W, Eck HP, Mihm S. HIV-induced cysteine deficiency and T-cell dysfunction—a rationale for treatment with *N*-acetylcysteine. *Immunol Today* 1992; **13**: 211–214.
191. Buttke TM, Sandstrom PA. Oxidative stress as a mediator of apoptosis. *Immunol Today* 1994; **15**: 7–10.
192. Adamson GM, Billings RE. Tumor necrosis factor induced oxidative stress in isolated mouse hepatocytes. *Arch Biochem Biophys* 1992; **294**: 223–229.
193. Satriano JA, Shuldiner M, Hora K, Xing Y, Shan Z, Schlondorff D. Oxygen radicals as second messengers for expression of the monocyte chemoattractant protein, JE/MCP-1, and the monocyte colony-stimulating factor, CSF-1, in response to tumor necrosis factor-alpha and immunoglobulin G. Evidence for involvement of reduced nicotinamide adenine dinucleotide phosphate (NADPH)-dependent oxidase. *J Clin Invest* 1993; **92**: 1564–1571.
194. Wong GH, Goeddel DV. Induction of manganous superoxide dismutase by tumor necrosis factor: possible protective mechanism. *Science* 1988; **242**: 941–944.
195. Kizaki M, Sakashita A, Karmakar A, Lin CW, Koeffler HP. Regulation of manganese superoxide dismutase and other antioxidant genes in normal and leukemic hematopoietic cells and their relationship to cytotoxicity by tumor necrosis factor. *Blood* 1993; **82**: 1142–1150.
196. Visner GA, Chesrown SE, Monnier J, Ryan US, Nick HS. Regulation of manganese superoxide dismutase: IL-1 and TNF induction in pulmonary artery and microvascular endothelial cells. *Biochem Biophys Res Commun* 1992; **188**: 453–462.
197. Melendez JA, Baglioni C. Reduced expression of manganese superoxide dismutase in cells resistant to cytolysis by tumor necrosis factor. *Free Radic Biol Med* 1992; **12**: 151–159.
198. Ishii Y, Partridge CA, Del Vecchio PJ, Malik AB. Tumor necrosis factor-alpha-mediated decrease in glutathione increases the sensitivity of pulmonary vascular endothelial cells to $H_2O_2$. *J Clin Invest* 1992; **89**: 794–802.
199. Kaslovsky RA, Gibbs L, Malik AB. Neutrophils from chronic granulomatous disease fail to increase endothelial permeability. *Am J Physiol* 1991; **261**: H1226–H1230.
200. Schreck R, Albermann K, Baeuerle PA. Nuclear factor kappa B: an oxidative stress-responsive transcription factor of eukaryotic cells. *Free Radic Res Commun* 1992; **17**: 221–237.
201. Baud L, Affres H, Perez J, Ardaillou R. Reduction in tumor necrosis factor binding and cytotoxicity by hydrogen peroxide. *J Immunol* 1990; **145**: 556–560.
202. Massague J. Transforming growth factor-alpha. A model for membrane-anchored growth factors. *J Biol Chem* 1990; **265**: 21393–21396.
203. Seckinger P, Isaaz S, Dayer JM. A human inhibitor of tumour necrosis factor alpha. *J Exp Med* 1988; **167**: 1511.
204. Smith CA, Davis T, Anderson D, *et al.* A receptor for tumor necrosis factor defines an unusual family of cellular and viral proteins. *Science* 1990; **248**: 1019–1023.
205. Schall TJ, Lewis M, Koller KJ, *et al.* Molecular cloning and expression of a receptor for human tumor necrosis factor. *Cell* 1990; **61**: 361–370.
206. Hsu KC, Chao MV. Differential expression and ligand binding properties of tumor necrosis factor receptor chimeric mutants. *J Biol Chem* 1993; **268**: 16430–16436.
207. Porteu F, Brockhaus M, Wallach D, Engelmann H, Nathan CF. Human neutrophil elastase releases a ligand-binding fragment from the 75-kDa tumor necrosis factor (TNF) receptor. Comparison with the proteolytic activity responsible for shedding of TNF receptors from stimulated neutrophils. *J Biol Chem* 1991; **266**: 18846–18853.
208. Descamps-Latscha B, Herbelin A. Long-term dialysis and cellular immunity. A critical survey. *Kidney Int* 1993; **43**: S135–S142.
209. Hakim RM, Breillatt J, Lazarus JM, Port FK. Complement activation and hypersensitivity reactions to dialysis membranes. *N Engl J Med* 1984; **311**: 878–882.
210. Nguyen AT, Lethias C, Zingraff J, Herbelin A, Naret C, Descamps-Latscha B. Hemodialysis membrane-induced activation of phagocyte oxidative metabolism detected *in vivo* and *in vitro* within microamounts of whole blood. *Kidney Int* 1985; **28**: 158–167.
211. Horl WH, Schaefer RM, Heidland A. Effect of different dialyzers on proteinases and proteinase inhibitors during hemodialysis. *Am J Nephrol* 1985; **5**: 320–326.
212. Vanholder R, Ringoir S, Dhondt A, Hakim R. Phagocytosis in uremic and hemodialysis: a prospective and cross sectional study. *Kidney Int* 1991; **39**: 320–327.
213. Descamps-Latscha B. The immune system in end-stage renal disease. *Curr Opin Nephrol Hypert* 1993; **2**: 883–891.
214. Seckinger P, Isaaz S, Dayer JM. Purification and biologic characterization of a specific tumor necrosis factor alpha inhibitor. *J Biol Chem* 1989; **264**: 11966–11973.
215. Descamps-Latscha B, Herbelin A, Nguyen AT, *et al.* Cytokines in the progression of renal failure. *Blood Purification* 1993; **11**: 287–288.
216. Pereira BJG, Shapiro L, King AJ, Falagas ME, Strom JA, Dinarello CA. Plasma levels of IL-1beta, TNF alpha and their specific inhibitors in undialyzed chronic renal failure, CAPD and hemodialysis patients. *Kidney Int* 1994; **45**: 890–896.
217. Baggiolini M, Clark-Lewis I. Interleukin-8, a chemotactic and inflammatory cytokine. *FEBS Lett* 1992; **307**: 97–101.
218. Deforge LE, Fantone JC, Kenney JS, Remick DG. Oxygen radical scavengers selectively inhibit interleukin 8 production in human whole blood. *J Clin Invest* 1992; **90**: 2123–2129.
219. Deforge LE, Preston AM, Takeuchi E, Kenney J, Boxer LA, Remick DG. Regulation of interleukin 8 gene expression by oxidant stress. *J Biol Chem* 1993; **268**: 25568–25576.
220. Samanta AK, Dutta S, Ali E. Modification of sulfhydryl groups of interleukin-8 (IL-8) receptor impairs binding of IL-8 and IL-8-mediated chemotactic response of human polymorphonuclear neutrophils. *J Biol Chem* 1993; **268**: 6147–6153.
221. Nakamura H, Yoshimura K, Jaffe HA, Crystal RG. Interleukin-8 gene expression in human bronchial epithelial cells. *J Biol Chem* 1991; **266**: 19611–19617.
222. Nakamura H, Yoshimura K, McElvaney NG, Crystal RG. Neutrophil elastase in respiratory epithelial lining fluid of individuals with cystic fibrosis induces interleukin-8 gene expression in a human bronchial epithelial cell line. *J Clin Invest* 1992; **89**: 1478–1484.
223. Mulligan MS, Jones ML, Bolanowski MA, *et al.* Inhibition of lung inflammatory reactions in rats by an anti-human IL-8 antibody. *J Immunol* 1993; **150**: 5585–5595.
224. Sacks T, Moldow CF, Craddock PR, Bowers TK, Jacob HS. Oxygen radicals mediate endothelial cell damage by complement-stimulated granulocytes. An *in vitro* model of immune vascular damage. *J Clin Invest* 1978; **61**: 1161–1167.
225. Ward PA. Mechanisms of endothelial cell injury. *J Lab Clin Med* 1991; **118**: 421–426.
226. Hiraishi H, Terano A, Razandi M, Sugimoto T, Harada T, Ivey KJ. Role of cellular superoxide dismutase against reactive oxygen metabolite injury in cultured bovine aortic endothelial cells. *J Biol Chem* 1992; **267**: 14812–14817.
227. Harlan JM, Levine JD, Callahan KS, Schwartz BR, Harker LA. Glutathione redox cycle protects cultured endothelial cells against lysis by extracellularly generated hydrogen peroxide. *J Clin Invest* 1984; **73**: 706–713.
228. Nathan CF, Arrick BA, Murray HW, Desantis NM, Cohn ZA. Tumor cell antioxidant defenses. Inhibition of the glutathione redox cycle enhances macrophage-mediated cytolysis. *J Exp Med* 1980; **153**: 766–782.
229. Charo IF, Yuen C, Perez HD, Goldstein IM. Chemotactic peptides modulate adherence of human polymorphonuclear leukocytes to monolayers of cultured endothelial cells. *J Immunol* 1986; **136**: 3412–3419.
230. Tonnesen MG, Anderson DC, Springer TA, Knedler A, Avdi N, Henson PM. Adherence of neutrophils to cultured human microvascular endothelial cells. Stimulation by chemotactic peptides and lipid mediators and dependence upon the Mac-1, LFA-1, p150,95 glycoprotein family. *J Clin Invest* 1989; **83**: 637–646.
231. Galanos C, Freudenberg MA. Bacterial endotoxins: biological properties and mechanisms of action. *Med Inflam* 1993; **2**: S11–S16.
232. Pohlman TH, Stanness KA, Beatty PG, Ochs HD, Harlan JM. An endothelial cell surface factor(s) induced *in vitro* by lipopolysaccharide, interleukin 1, and tumor necrosis factor-alpha increases neutrophil adherence by a CDw18-dependent mechanism. *J Immunol* 1986; **136**: 4548–4553.
233. Gamble JR, Harlan JM, Klebanoff SJ, Vadas MA. Stimulation of the adherence of neutrophils to umbilical vein endothelium by human recombinant tumor necrosis factor. *Proc Natl Acad Sci USA* 1985; **82**: 8667–8671.
234. Pober JS, Lapierre LA, Stolpen AH *et al.* Activation of cultured human endothelial cells by recombinant lymphotoxin: comparison with tumor necrosis factor and interleukin 1 species. *J Immunol* 1987; **138**: 3319–3324.
235. Lawrence MB, McIntyre LV, Eskin SG. Effect of flow on polymorphonuclear leukocyte/endothelial cell adhesion. *Blood* 1987; **70**: 1284–1290.
236. Harlan JM. Leukocyte-endothelial interactions. *Blood* 1985; **65**: 513–525.
237. Von Andrian UH, Chambers JD, McEvoy LM, Bargatze RF, Arfors KE, Butcher EC. Two-step model of leukocyte-endothelial cell interaction in inflammation: distinct roles for LECAM-1 and the leukocyte beta 2 integrins *in vivo*. *Proc Natl Acad Sci USA* 1991; **88**: 7538–7542.
238. Lawrence MB, Springer TA. Leukocytes roll on a selectin at physiologic flow rates: distinction from and prerequisite for adhesion through integrins. *Cell* 1991; **65**: 859–873.
239. Smith CW, Kishimoto TK, Abbassi O, *et al.* Chemotactic factors regulate lectin adhesion molecule 1 (LECAM-1)-dependent neutrophil adhesion to cytokine-stimulated endothelial cells *in vitro* (published erratum appears in *J Clin Invest* 1991; **87(5)**: 1873). *J Clin Invest* 1991; **87**: 609–618.

240. Schleiffenbaum B, Moser R, Patarroyo M, Fehr J. The cell surface glycoprotein Mac-1 (CD11b/CD18) mediates neutrophil adhesion and modulates degranulation independently of its quantitative cell surface expression. *J Immunol* 1989; **142**: 3537–3545.
241. Newman PJ, Berndt MC, Gorski J, *et al.* PECAM-1 (CD31) cloning and relation to adhesion molecules of the immunoglobulin gene superfamily. *Science* 1990; **247**: 1219–1222.
242. Muller WA, Weigl SA, Deng X, Phillips DM. PECAM-1 is required for transendothelial migration of leukocytes. *J Exp Med* 1993; **178**: 449–460.
243. Bradley JR, Johnson DR, Pober JS. Endothelial activation by hydrogen peroxide. Selective increases of intercellular adhesion molecule-1 and major histocompatibility complex class I. *Am J Pathol* 1993; **142**: 1598–1609.
244. Sellak H, Franzini E, Hakim J, Pasquier C. Reactive oxygen species rapidly increase endothelial ICAM-1 ability to bind neutrophils without detectable upregulation. *Blood* 1994 (in press).
245. Patel KD, Zimmerman GA, Prescott SM, McEver RP, McIntyre TM. Oxygen radicals induce human endothelial cells to express GMP-140 and bind neutrophils. *J Cell Biol* 1991; **112**: 749–759.
246. Palluy O, Morliere L, Gris JC, Bonne C, Modat G. Hypoxia/reoxygenation stimulates endothelium to promote neutrophil adhesion. *Free Radic Biol Med* 1992; **13**: 21–30.
247. Allen RC, Stevens DL. The circulating phagocyte reflects the in vivo state of immune defense. *Curr Opin Infect Dis* 1992; **5**: 389–398.
248. Tosi MF, Berger M. Functional differences between the 40 kDa and 50 to 70 kDa IgG Fc receptors on human neutrophils revealed by elastase treatment and antireceptor antibodies. *J Immunol* 1988; **141**: 2097–2103.
249. Fearon DT, Collins LA. Increased expression of C3b receptors on polymorphonuclear leukocytes induced by chemotactic factors and by purification procedures. *J Immunol* 1983; **130**: 370–375.
250. Neuman E, Huleatt JW, Jack RM. Granulocyte-macrophage colony stimulating factor increases synthesis and expression of CR1 and CR3 by human peripheral blood neutrophils. *J Immunol* 1990; **145**: 3325–3332.
251. Tielemans CL, Delville JP, Husson CP, *et al.* Adhesion molecules and leukocyte common antigen on monocytes and granulocytes during hemodialysis. *Clin Nephrol* 1993; **39**: 158–165.
252. Berger M, Sorensen RU, Tosi MF, Dearborn DG, Döring G. Complement receptor expression on neutrophils at an inflammatory site, the *Pseudomonas*-infected lung in cystic fibrosis. *J Clin Invest* 1989; **84**: 1302–1313.
253. Remold-O'Donnell E, Davis AE, Kenney D, Bhaskar KR, Rosen FS. Purification and chemical composition of gpL115, the human lymphocyte surface sialoglycoprotein that is defective in Wiskott–Aldrich syndrome. *J Biol Chem* 1986; **261**: 7526–7530.
254. Carlsson SR, Sasaki H, Fukuda M. Structural variations of O-linked oligosaccharides present in leukosialin isolated from erythroid, myeloid, and T-lymphoid cell lines. *J Biol Chem* 1986; **261**: 12787–12795.
255. Campanero MR, Pulido R, Alonso JL, *et al.* Down-regulation by tumor necrosis factor-alpha of neutrophil cell surface expression of the sialophorin CD43 and the hyaluronate receptor CD44 through a proteolytic mechanism. *Eur J Immunol* 1991; **21**: 3045–3048.
256. Rieu P, Porteu F, Bessou G, Lesavre P, Halbwachs-Mecarelli L. Human neutrophils release their major membrane sialoprotein, leukosialin (CD43), during cell activation. *Eur J Immunol* 1992; **22**: 3021–3026.
257. Bazil V, Strominger JL. CD43, the major sialoglycoprotein of human leukocytes, is proteolytically cleaved from the surface of stimulated lymphocytes and granulocytes. *Proc Natl Acad Sci USA* 1993; **90**: 3792–3796.
258. Francis JW, Todd RD, Boxer LA, Petty HR. Sequential expression of cell surface C3bi receptors during neutrophil locomotion. *J Cell Physiol* 1989; **140**: 519–523.
259. Woodman RC, Reinhardt PH, Kanvar S, Johnston FL, Kubes P. Effects of human neutrophil elastase on neutrophil function *in vitro* and in inflamed microvessels. *Blood* 1993; **82**: 2188–2195.

ACKNOWLEDGEMENT. We thank Drs Joëlle Gabay Lise Halbwachs-Mécarelli, Patricia Lemarchand and Catherine Pasquier, for helpful comments concerning this review and the Association Française de Lutte contre la Muscoviscidose (AFLM) for supporting the PhD thesis of Véronique Witko-Sarsat by the AFLM Research Grant.

**Received 6 April 1994; accepted in revised form 12 April 1994**
